# Long read and single molecule DNA sequencing simplifies genome assembly and TAL effector gene analysis of *Xanthomonas translucens*

**DOI:** 10.1186/s12864-015-2348-9

**Published:** 2016-01-05

**Authors:** Zhao Peng, Ying Hu, Jingzhong Xie, Neha Potnis, Alina Akhunova, Jeffrey Jones, Zhaohui Liu, Frank F. White, Sanzhen Liu

**Affiliations:** Department of Plant Pathology, Kansas State University, Manhattan, KS USA; Department of Horticulture, Forestry and Recreation resources, Kansas State University, Manhattan, KS USA; Department of Plant Pathology, University of Florida, Gainesville, FL USA; Department of Plant Pathology, North Dakota State University, Fargo, ND USA

**Keywords:** Bacterial leaf streak, *X. translucens*, PacBio, TAL effectors

## Abstract

**Background:**

The species *Xanthomonas translucens* encompasses a complex of bacterial strains that cause diseases and yield loss on grass species including important cereal crops. Three pathovars, *X. translucens* pv*. undulosa*, *X. translucens* pv*. translucens* and *X. translucens* pv*.cerealis*, have been described as pathogens of wheat, barley, and oats. However, no complete genome sequence for a strain of this complex is currently available.

**Results:**

A complete genome sequence of *X. translucens* pv*. undulosa* strain XT4699 was obtained by using PacBio long read, single molecule, real time (SMRT) DNA sequences and Illumina sequences. Draft genome sequences of nineteen additional *X. translucens* strains, which were collected from wheat or barley in different regions and at different times, were generated by Illumina sequencing. Phylogenetic relationships among different *Xanthomonas* strains indicates that *X. translucens* are members of a distinct clade from so-called group 2 xanthomonads and three pathovars of this species, *undulosa*, *translucens* and *cerealis*, represent distinct subclades in the group 1 clade. Knockout mutation of type III secretion system of XT4699 eliminated the ability to cause water-soaking symptoms on wheat and barley and resulted in a reduction in populations on wheat in comparison to the wild type strain. Sequence comparison of *X. translucens* strains revealed the genetic variation on type III effector repertories among different pathovars or within one pathovar. The full genome sequence of XT4699 reveals the presence of eight members of the Transcription-Activator Like (TAL) effector genes, which are phylogenetically distant from previous known TAL effector genes of group 2 xanthomonads. Microarray and qRT-PCR analyses revealed TAL effector-specific wheat gene expression modulation.

**Conclusions:**

PacBio long read sequencing facilitates the assembly of *Xanthomonas* genomes and the multiple TAL effector genes, which are difficult to assemble from short read platforms. The complete genome sequence of *X. translucens* pv*. undulosa* strain XT4699 and draft genome sequences of nineteen additional *X. translucens* strains provides a resource for further genetic analyses of pathogenic diversity and host range of the *X. translucens* species complex. TAL effectors of XT4699 strain play roles in modulating wheat host gene expressions.

**Electronic supplementary material:**

The online version of this article (doi:10.1186/s12864-015-2348-9) contains supplementary material, which is available to authorized users.

## Background

Bacterial pathogens of the genus *Xanthomonas* cause disease symptoms in a wide range of plant species, including many economically important cereal crops [1]. The species *X. translucens* represents a complex of strains that are pathogenic on various members of the *Poaceae*, including wheat, barley, oat, rye and other grass species. Bacterial leaf streak (BLS) and black chaff symptoms in the grain spikes on wheat are caused by *X. translucens* pv. *undulosa* strains. Outbreaks of BLS occur sporadically in central Great Plains and are associated with relatively warm and humid conditions, although the disease has been prevalent in recent recurrent years in the northern Great Plains [2]. *X. translucens* strains have been classified by pathogenicity types and DNA fingerprinting technologies [3]. Strains causing disease symptoms on barley and wheat are named as *X. translucens* pv. *undulosa*, while strains only pathogenic on barley are called *X. translucens* pv. *translucens* [4]. Although some strains of *X. translucens* pv. *cerealis* behave similarly as *X. translucens* pv. *undulosa* in pathogenicity types, they are distinguishable by DNA fingerprinting [3]. Phylogenic analyses of various *X. translucens*, do not align with the pathovar designations, and clarifications await genomic analyses on larger strain collections. In addition, many strains that were isolated from other species, often have been reported to cause disease symptoms on wheat [3]. For example, thirty-three bacterial strains isolated from diseased ornamental asparagus were identified as *X. translucens* pv. *undulosa* using DNA fingerprinting and cross inoculation [5].

Next-generation sequencing technologies have made transformational changes over the Sanger sequencing by improving throughput and reducing cost [6, 7]. The draft genome sequences provide valuable information on major genome contents and enable genome comparison among strains of interest [8, 9]. Currently, draft genome sequences are available for four strains from the *X. translucens* group. Draft genomic data for *X. translucens* pv. *undulosa* strain DAR61454, *X. translucens* pv. *transluencs* strain DSM18974, *X. translucens* pv. *cerealis* CFBP2541, and *X. translucens* pv. *graminis* ART-Xtg29 were generated by using Illumina or Roche 454 short read sequencing platforms [8–10]. At the same time, genome assemblies based on Illumina and Roche 454 sequencing are fragmented, and most assemblies failed to assemble complex repetitive sequences, including Transcription Activator-Like (TAL) effector genes, which occur in multiple gene copies and contain multiple simple near-perfect repeats within each gene. TAL effector genes typically have highly conserved N- and C-terminal sequences, and harbor 12.5–28.5 units of 102 or 105 bp repeats in the central regions [1]. Recently, a single-molecule real-time (SMRT) sequencing technology was developed by Pacific Bioscience (PacBio) and produces long sequence reads with no obvious sequencing biases and may allow better resolution of long repetitive DNA features in a genome. Due to a high error rate of PacBio reads, a high sequencing depth (e.g., 50× or higher) is usually required for a high-quality *de novo* assembly [11–13].

In this study, the complete genome sequence of *X. translucens* pv. *undulosa* strain XT4699 was generated by using high-depth PacBio and Illumina data. The genome was compared to draft genomic sequences of nineteen additional *X. translucens* strains that were derived by Illumina. Comparisons of TAL effectors from three *X. translucens* strains and their relationship with TAL effectors from other *Xanthomonas* species were also performed, and evidence for TAL effector-dependent effects on host gene expression was assessed by microarray and qRT-PCR analyses.

## Results

### Complete genome assembly of XT4699

**Fig. 1:**
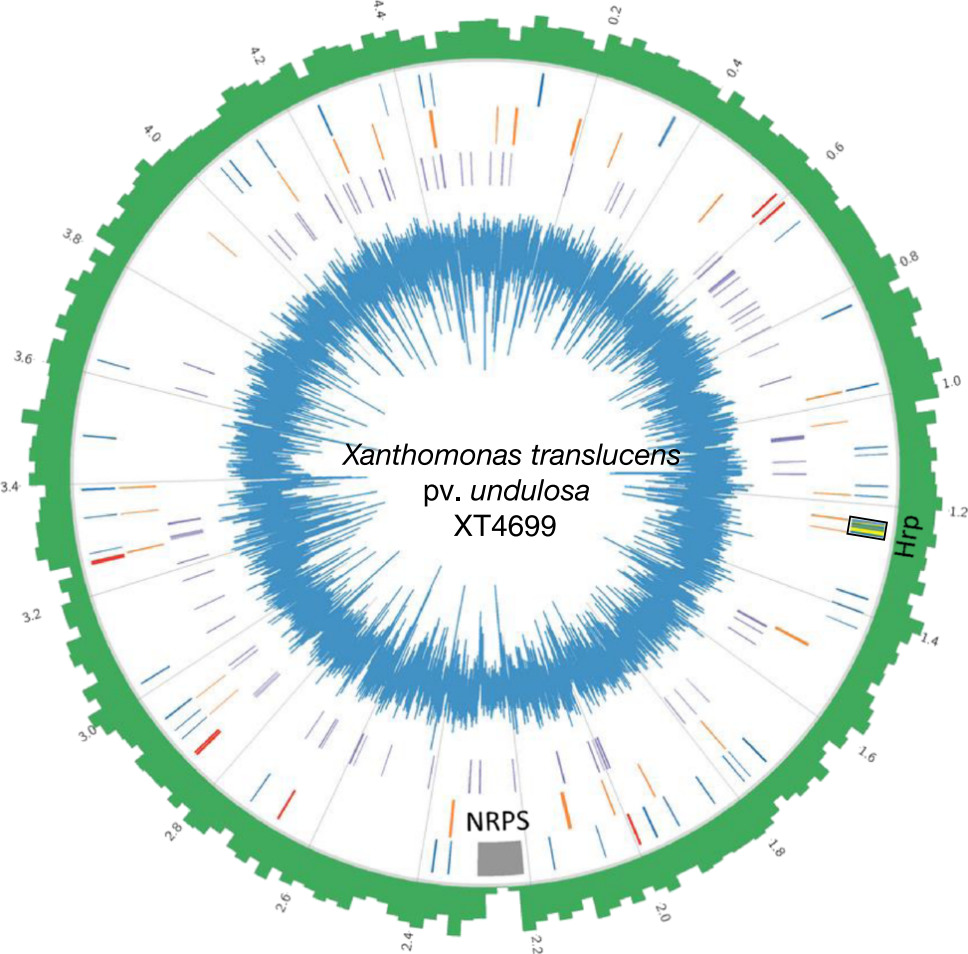
Circular representation of *X. translucens* pv. *undulosa* strain XT4699 genome. The outer histogram (*green*) indicates gene density, while the inner histogram (*blue*) shows the GC content. From the outside to inside, layer 1 indicates the location of, the NRPS genes (*grey*), the T3SS genes cluster (*yellow in a black rectangle*), the genes with PIPs (*blue*), and TAL effector genes (*red*). In layer 2, all non-TAL type III effector genes are marked by orange color, and, in layer 3, IS elements are shown in purple

Illumina 2x250 bp MiSeq data for XT4699 provided approximately 60x coverage, comprising 267.5 Mbp total sequencing bases. The assembly *via* SOAPdenov2 resulted in 547 contigs of at least 400 bp in length. The total assembly length was 4.4 Mb and the N50 equals to 15,114 bp. Two PacBio SMRT cells of 10-kb library preparation for XT4699 generated 114,394 raw reads and a total of 446 Mb. The average length of reads was 3,898 bp. The reads were assembled into two long contigs using an optimized assembly pipeline, HGAP2 [12]. The longest contig is 4,357,621 bp in length. Two contigs were merged into a single contig *via* combining with Illumina assembled contigs using the minimus2 module in AMOS (sourceforge.net/apps/mediawiki/amos). Alignments of PacBio raw reads to the resulting single contig identified multiple PacBio reads spanning the junction of two original Pacbio contigs, providing additional evidence for the contig merging. Two ends of the resulting single contig shared 8,439 bp with 99.33 % identity. The contig was circularized after removing the sharing sequence at one end. The circularized assembly draft was subjected to an additional two rounds of error correction using the PacBio resequencing pipeline that includes the error correction module [12]. Consequently, a single finished genome sequence (*N* = 4,561,137 bp) was obtained (Fig. 1).

To assess the assembly quality, Illumina reads were mapped to the assembled sequence. 99.53 reads can be mapped and 95.46 % are uniquely mapped with the stringent mapping criteria (see Methods). Respectively, 99.99 and 99.44 % of the assembled genome was covered by mapping reads and uniquely mapped reads. Based on the alignments, no mismatches were identified between reads and the assembled genome (see Methods), indicating the high quality of the assembly sequence. The Illumina-only assembly contigs were also aligned *via* nucmer [14] to the final assembly, and approximately 97 % of the final assembly was covered by the Illumina contigs. Of the 547 contigs, 98.54 % (539/547), accounting for 99.77 % total contig sequence, can be aligned to the sequence with four exceptions (see Methods). Four single nucleotide variants, including two substitutions and two INDELs (insertion-and-deletions), were identified between the Illumina assembly contigs and the final assembly (Additional file 1: Table S1). To assess the quality of the assembly in the repetitive regions, the eight TAL effector genes were cloned by high fidelity PCR and sequenced using the standard Sanger protocol. All the Sanger sequences were perfectly matched to the assembled genome sequence with 100 % identity (Additional file 2: Table S2).

### XT4699 genome content

The XT4699 genome is a single circular chromosome (4,561,137 bp) with an overall G + C content of 68.1 %. The complete genome, as annotated by the NCBI Prokaryotic Genome Automatic Annotation Pipeline (PGAAP), contains 3,528 coding DNA sequence (CDS) genes, 94 pseudogenes, and 54 genes with frameshift mutation that alters open reading frame. XT4699 has 2 ribosomal RNA operons and 54 tRNA genes. The genome contains 74 predicted insertion sequence (IS) elements and 56 partial IS elements using the ISfinder program (Table 1). The genome encodes three long non-ribosomal peptide synthesis (NRPS) proteins of 4827, 7451, and 6419 amino acids, respectively, in a cluster of 80 kb regions flanked by IS elements (Fig. 1). A plant inducible promoter (PIP) element is present in 49 genes, including some type III secretion system (Hrp) and effector genes (Fig. 1, Additional file 3: Table S3).

**Table 1:** Genome features of *X. translucens* pv. *undulosa* strain XT4699

Features	Values
Genome size (bp)	4,561,137
GC content (%)	68.1
Number of predicted CDS genes	3528
Pseudogenes	94
Genes with frameshift	54
TAL effector genes	8
Non-TAL T3E genes	32
rRNA operons	2
tRNA	54
CRISPR array	Not detected
PIP genes	49
Insertion sequence elements (complete/partial)	74/56

The XT4699 genome consists of 4.3 %, or approximately 0.2 Mb, of repetitive sequence. Among 252 genomic repetitive regions, 163 repeats are less than 1 kb and 89 repeats are longer than 1 kb. The longest repeats, located around FD63_00725 and FD63_01915 ribosome RNA genes, are approximately 6 kb with the 99.98 % identity between two copies. Eleven pairs of tandem repeats were identified, ranging in size from 107 to 537 bp. In terms of copy number, 42.5 of the repeats have two copies and 57.5 % repeats have three or more copies. Tandem repeats and high copy repeats (>2) increase the complexity of the assembly. In the Illumina-based assembly, more than 90 % of the tandem repeats occurred at assembly gaps, even when relatively short (115–375 bp), and the vast majority of high-copy repeats were not resolved. With one exception, repetitive regions with length greater than 500 bp were not resolved in the Illumina-based assembly. Overall, 40 % of the gaps in the Illumina-only assembly were located at repetitive regions.

The overall GC percentage of the XT4699 final assembly is 68.1 %, while the overall GC percentage of the Illumina-only assembly is 67.2 %, indicating GC-rich regions are not equally represented in the Illumina-based assembly. To assess Illumina and PacBio sequence results for GC-rich regions, the final assembly genome was scanned with non-overlapping 200 bp windows, and the GC% and sequencing coverage of both Illumina and PacBio sequences were determined at each window. Scatter plots showed a GC bias in sequencing coverage of Illumina but not PacBio, which at least partially explains high variation of Illumina sequencing coverage and uniform distribution of PacBio sequencing reads across the genome (Additional file 4: Figure S1). The vast majority (50/55) of extremely high GC regions, which exhibit > 80 % GC content, overlap with assembly gaps in the Illumina-only assembly. Most gaps on GC rich regions are very small (<100 bp). The flanking contigs of many of these gaps have overlaps, but the overlapping sequences between two adjacent contigs are not long enough to join them. On the contrary, gaps caused by repeats are generally longer than GC-rich gaps. The sizes of repeat-induced gaps are linearly correlated with repeat sizes (Additional file 5: Figure S2). Regardless of gaps in the Illumina-only assembly, the contig sequences exhibit high accuracy when compared with the final assembly (Additional file 4: Figure S1).

### Phylogenetic relationship and pathogenicity types of *X. translucens* strains

**Fig. 2:**
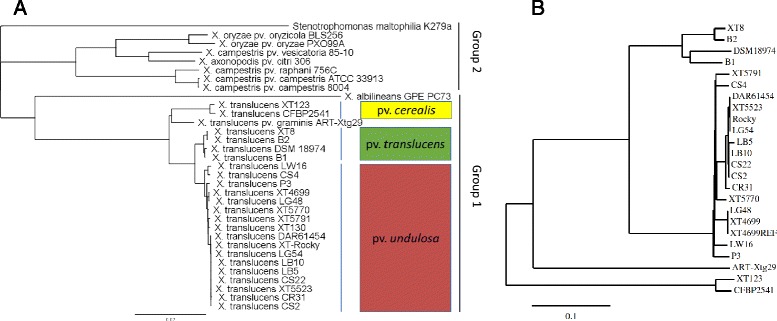
Phylogenetic trees. **a** Phylogenetic tree in the genus of *Xanthomonas* using MLSA. Nucleotide sequence of four concatenated housekeeping genes (*DnaK*, *gyrB*, *GroEL* and *RecA*) was extracted from the draft or complete genomes. The tree was constructed by applying all the concatenated sequences into Geneious software Version 6 with the Tamura-Nei genetic distance model and the Neighbor-joining method, with *Stenotrophomonas maltophilia* K279a as an outgroup. The scale bar indicates number of nucleotide substitutions per site. The dark red represents *undulosa* pathovars, the green color indicates the *translucens* pathovar and the yellow color represents the *cerealis* pathovars; **b** Phylogenetic tree of *X. translucens* strains using whole-genome discovery of SNP. The scale bar indicates number of nucleotide substitutions per site. Details are described in Methods

Genome assembly using Illumina 2x250 bp reads for other 19 *X. translucens* strains was performed with contigs having 70–200x coverage. The N50, assembled genome size, maximum contig length, and contig number of each assembly are shown in Table 2. Consistent with the classification results from previous studies [8, 9], multilocus sequence analysis (MLSA) revealed the *X. translucens* strains were closer to *X. albilineans* in xanthomonad group 1 than *X. oryzae* and *X. campestris* in group 2 (Fig. 2a). The *X. translucens* pv. *undulosa* strains, XT4699, XT-Rocky, LB10, P3, LG48, and DAR61454, which are all virulent on both wheat and barley, were separated from the *X. translucens* pv. *translucens* strains XT8, B1, B2 and DSM18974, which are only virulent on barley. In addition, the above strains were distinguishable from the *X. translucens* pv. *cerealis* strains CFBP2541 and XT123, and *X. translucens* pv. *graminis* ART-Xtg29, which is consistent with previous phylogenetic classifications based on AFLP analysis [3]. The phylogenetic tree developed by a whole genome SNP comparison further corroborated MLSA results, providing higher resolution of evolutionary relationship among closely related strains (Fig. 2b, Additional file 6: Table S4). Strains LG54, XT5523, XT-Rocky, CS2, CS22, LB10, and DAR61454 are highly related, although they were isolated from different geographic origins and at different times (Table 2). The strains have near identical housekeeping genes, ranging from 2 to 17 SNPs in individual strains out of a total of 9,836 SNPs among the strains (Additional file 6: Table S4). XT4699 and LG48 are another pair of highly related strains on the basis of MLSA and the whole genome SNP comparison (Fig. 2).

**Table 2:** Information of bacterial isolation, pathogenicity type and genome assembly statistics for *X. translucens* strains

Strain name	Isolation			Pathogenicity type^c^	Assembly statistics			
	Year	Host origin	Place		Contig number	Genome size (bp)	N50 (bp)	Maximum contig length (bp)
Strains used in this study								
XT-4699^a^	1999	Wheat	KS,USA	A	1	4,561,137	NA	NA
XT-Rocky	2009	Wheat	KS,USA	A	521	4,459,068	21,643	85,632
XT8	1942	Barley	Canada	B	152	4,617,556	79,563	279,321
XT123	1952	Barley	Canada	A	344	4,284,749	28,687	135,676
XT130	1939	NA	Canada	A	329	4,654,290	95,229	230,109
XT5523^b^	1966	Wheat	Canada	A	330	4,665,768	111,158	270,930
XT5770	NA	NA	Canada	A	533	4,617,837	27,152	149,044
XT5791	1969	Wheat	Canada	A	738	4,719,363	62,926	185,369
B1	2013	Barley	ND,USA	B	642	4,824,098	28,186	96,565
B2	2013	Barley	ND,USA	B	283	4,503,259	38,929	154,569
P3	2009	Wheat	ND,USA	A	161	4,522,131	78,928	240,099
LW16	2009	Wheat	ND,USA	A	325	4,600,125	33,769	145,918
LB5	2009	Wheat	ND,USA	A	434	4,766,161	111,172	269,877
LB10	2009	Wheat	ND,USA	A	157	4,543,985	98,403	211,728
LG48	2009	Wheat	ND,USA	A	293	4,486,555	38,250	111,281
LG54	2009	Wheat	ND,USA	A	213	4,623,672	123,210	274,463
CS2	2009	Wheat	ND,USA	A	285	4,722,832	85,714	207,734
CS22	2009	Wheat	ND,USA	A	129	4,605,395	95,219	274,395
CR31	2009	Wheat	ND,USA	A	386	4,720,715	62,461	158,913
CS4	2009	Wheat	ND,USA	A	498	4,779,534	37,460	107,554
Strains from references								
CFBP2541^b^	1941	Bromegrass	USA	A	31	4,515,938	1,399,657	1,809,000
DSM18974^b^	1933	Barley	MN,USA	B	551	4,463,577	13,041	55,714
DAR61454	1988	Wheat	Australia	A	404	4,452,091	27,210	121,856
ART-Xtg29	NA	Forage grass	Switzerland	C	788	4,100,864	8376	37,754

NA means not available or not applicable

^a^indicates genome assembly with PacBio long-read sequencing

^b^Type strains

^c^Strains virulent on wheat and barley are classified as pathogenicity type A, strains virulent only on barley are considered as type B and strains virulent on forage grass are assigned to type C

Based on previous pathogenicity tests, *X. translucens* strains have been grouped into type A, pathogenic on both wheat and barley, type B, only pathogenic on barley, and type C, not pathogenic on either [3]. Though strains from *X. translucens* pv. *undulosa* and *X. translucens* pv. *cerealis* are classified as pathogenicity type A, they are distant in the phylogenetic relationship (Fig. 2). Here, cultivars other than ‘Alondra’ wheat and ‘Corona’ barley were tested to determine possible pathogenicity differences between strains from each subgroup. Three representative strains, XT4699 (*X. translucens* pv. *undulosa*), XT8 (*X. translucens* pv. *translucens*) and XT123 (*X. translucens* pv. *cerealis*), from each subgroup were selected and inoculated on ‘Chinese Spring’, ‘Jagger’, ‘Hope’ and ‘Canthatch’ hexaploid wheat, one *Triticum turgidum* wheat (accession number 107 in WGRC at Kansas State University), ‘KS Southeast’ and ‘Morex’ barley. XT4699 induced water-soaking symptoms on ‘Chinese Spring’, ‘Jagger’, ‘Hope’, ‘Canthatch’ wheat and ‘KS Southeast’ barley, while inducing mixed symptoms of chlorosis and water soaking on ‘Morex’ barley. XT123 only triggered chlorosis symptoms at 4 DPI on ‘Chinese Spring’, ‘Jagger’, ‘Hope’ wheat and ‘KS Southeast’, and ‘Morex’ barley (Additional file 7: Figure S3). Though XT123 could cause water-soaking on ‘Canthatch’ and *Triticum turgidum* wheat cultivars, the strain exhibited different lesion symptoms from XT4699 (Additional file 7: Figures S3F and S3G). XT8 caused strong water soaking symptoms on ‘KS Southeast’ barley and less severe water soaking lesions on ‘Morex’ barley, while only inducing chlorosis on all the wheat cultivars (Additional file 7: Figure S3).

### CRISPR clusters are present in most *X. translucens* strains

Clustered regularly interspaced short palindromic repeat (CRISPR) and CRISPR-associated genes (*Cas*) comprise an adaptive bacterial immune system against foreign DNA [15]. The spacer sequences between repeats in the CRISPR loci typically correspond to sequences of perfect copies, called proto-spacer, of sequences in foreign DNA elements and direct the their cleavage [16]. The sequencing and monitoring of repeat and spacer array of CRISPR in strains provides insight into the coevolution relationship between strains and invader phage DNA. The DNA elements of CRISPR loci contain a record of past immunity events and can reveal relationships between closely related strains and populations [17, 18].

Genomic sequence data of the *X. translucens* strains in this study and previously sequenced strains revealed CRISPR loci are present in most *X. translucens* strains, except for XT4699, LG48, and XT123. If present, only one CRISPR locus is found in each strain. The CRISPR loci are often flanked by transposon gene elements. Annotation of CRISPR *Cas* genes of all *X. translucens* strains and *X. oryzae* pv. *oryzae* strain PXO99 indicates that the strains belong to the Type I-C (Dvulg or CASS1) in the classification of CRISPR-Cas systems [19]. The phylogenetic relationship, based on the nucleotide sequences of *Cas* gene clusters, among these strains is different from the MLSA and whole genome SNP comparisons (Fig. 2, Additional file 8: Figure S4), suggesting the evolution of CRISPR-Cas system is somewhat independent of genome evolution. LW16 and CS4 strains are special variants in this subtype, harboring two *Cas3* helicase genes, while other strains only have one. The sequence of direct repeats in the CRISPR loci is conserved among *X. oryzae* pv. *oryzae* strain PXO99 and *X. translucens* strains, although CRISPR *Cas* loci are distinct.

**Fig. 3:**
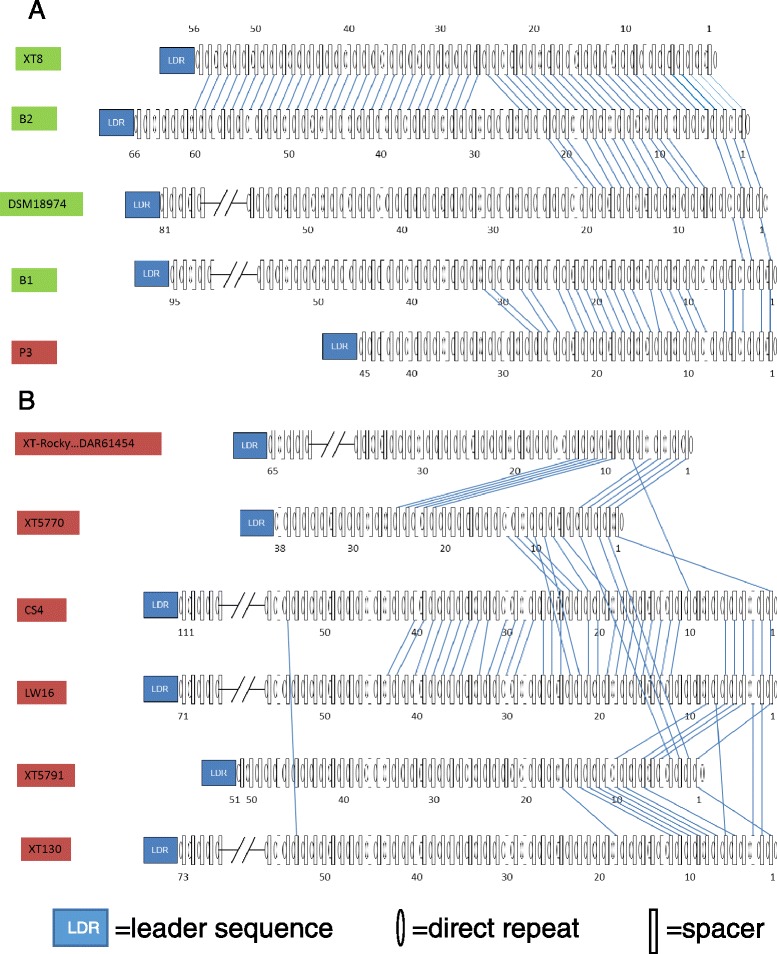
Spacer comparison among different *X. translucens* strains. The leader sequence, spacer and direct repeat sequence are analyzed at http://crispr.u-psud.fr/Server/. The identical spacer elements are linked together by blue lines. The strains of *undulosa* pathovar and *translucens* pathovar are marked with dark red and green, respectively. There is no identical spacer between panel (**a**) and (**b**). The XT-Rocky…DAR61454 represents 9 strains of LG54, XT5523, XT-Rocky, CR31, CS2, CS22, LB5, LB10 and DAR61454, which share the same *Cas* genes, leader sequence, direct repeat and spacer arrays

The comparison of spacer elements among strains revealed that *X. translucens* pv. *undulosa* and *X. translucens* pv. *translucens* have distinct spacers, with the exception that *X. translucens* pv. *undulosa* strain P3. P3 shares twenty-three identical spacers with *X. translucens* pv. *translucens* strain B1, while harboring no identical spacer element with any other *X. translucens* pv. *undulosa* strain. Strains P3 and B1, though distinct in phylogeny and pathogenicity types, may have experienced an overlapping history of immunity or, alternatively, the strains have acquired the CRISPR elements by lateral gene transfer. *X. translucens* pv. *translucens* strains XT8 and B2 have closely related CRISPR *Cas* loci and share fifty-five identical spacers (Fig. 3a, Additional file 8: Figure S4). LW16 and CS4 share thirty-two identical spacers, the highest observed among *X. translucens* pv. *undulosa* strains (Fig. 3b). Although the older spacer elements in the *X. translucens* strains are shared among some strains, the most recently acquired spacer sequences are unique to each strain. (Fig. 3). However, nine strains, XT-Rocky (collected from KS in 2009), XT5523 (collected from Canada in 1966), LG54, CR31, CS2, CS22, LB5, LB10 (collected from ND in 2009) and DAR61454 (collected from Australia in 1988), share identical elements of *Cas* cluster, repeats, and spacer array (Fig. 3b), implying that CRISPR *Cas* loci can be maintained over decades. The spacer sequences of *X. translucens* pv. *cerealis* CFBP2541 and *X. translucens* pv. *graminis* ART-Xtg29 strains were not detected in any other *X. translucens* strains.

### The type III secretion system of *X. translucens*

**Fig. 4:**
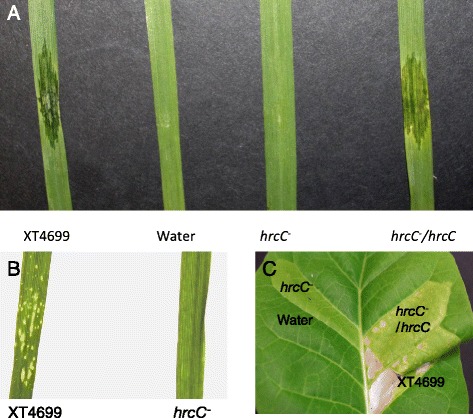
Phenotypic difference between XT4699 and *hrcC* ^−^ mutant. Panel **a**, third leaves of one-month-old Chinese Spring wheat plants were separately inoculated with XT4699, water, *hrcC* ^−^ and complementation strain of *hrcC* ^−^ by needleless syringe. The photo was taken at 4DPI. **b** WT and *hrcC* ^−^ (OD600 = 0.2) strains were coated with 0.02 % Silwet L-77 and applied for the dip inoculation assays. Second leaves of 14-day-old Chinese Spring wheat plants were used for experiment. The photo was taken at 8DPI. C, the same inoculum, as in panel **a**, was infiltrated into leaves of two-month-old KY-14 tobacco plants. It was photographed at 8DPI

Comparative genomics have previously revealed extensive divergence of the type III secretion system (T3SS) between group 1 and group 2 of *Xanthomonas* species [9]. *X. albilineans* does not possess the T3SS, which is usually present and crucial for pathogenicity in group 2 *Xanthomonas* species [20]. Disruption of the T3SS in *X. translucens* pv. *graminis* strain ART-Xtg29 did not eliminate disease symptoms, and the survival of T3SS mutants inside of plant tissue was reported to be unaffected when compared to the wild type strain up to 14 days post infection (DPI) [9]. The T3SS gene clusters in XT4699 and ART-Xtg29 strains are syntenic but have lower sequence identity in T3SS proteins compared to the identity between *X. translucens* pv. *undulosa* strains (Additional file 9: Figure S5). An insertion mutant of the conserved component gene *hrcC* in XT4699 (XT4699*hrcC*^*−*^) resulted in loss of disease symptoms on ‘Chinese Spring’ wheat on the basis of water-soaking lesions, which were not visible by 10 DPI in the mutant, while wild type XT4699 caused extensive water-soaked lesion starting at 3 DPI (Fig. 4a). Re-introduction of the native *hrcC* gene into the mutant strain fully complemented the loss of disease symptoms (Fig. 4a). Using an alternative dip inoculation assay, no disease was present 10 DPI for XT4699*hrcC*^*−*^, while the disease spots appeared at 4–5 DPI for the XT4699 (Fig. 4b). The ability to cause disease symptoms on barley was also lost in XT4699*hrcC*^*−*^. Two different barley cultivars, ‘KS Southeast’ and ‘Morex’, were used in the inoculation assays. XT4699 formed sharply delineated water-soaked lesions and a mixed symptom of chlorosis and water soaking at 3 DPI on ‘KS Southeast’ and ‘Morex’, respectively, while XT4699*hrcC*^*−*^ failed to develop symptoms on either cultivar (Additional file 7: Figures S3B and S3C). In addition, XT4699*hrcC*^*−*^ failed to trigger a hypersensitive reaction (HR) and strong chlorosis on non-host KY14 tobacco, while the XT4699 and the *hrcC*^*−*^ complementation strains did induce an HR (Fig. 4c). Bacterial population assays in ‘Chinese Spring’ wheat indicated that the XT4699*hrcC*^*−*^ population was lower by approximately 450 fold in comparison to XT4699 at 6DPI (Additional file 10: Figure S6).

### Type III effector gene content

**Fig. 5:**
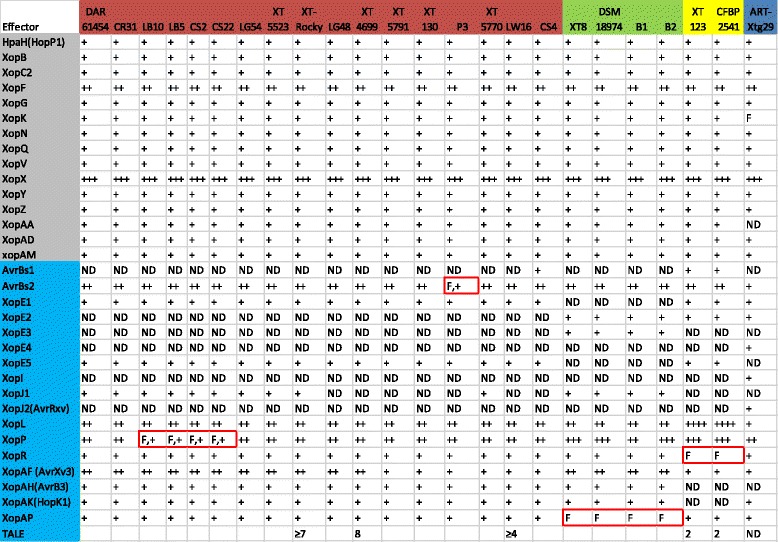
Comparison of Type III effector repertories among four pathovars of *X. translucens*. Fifteen effectors (from HapH to XopAM), marked with gray color, are predicted to be conserved among *undulosa*, *translucens* and *cerealis* pathovars, while seventeen remaining effectors (from AvrBs1 to XopAP), marked with blue color, are shown to be variable among strains. Single copy of an effector is indicated by ‘+’ and multiple copies are indicated by multiple ‘+’. An effector that is not detected is indicated as ‘ND’. The frameshift mutation in an effector gene is marked with ‘F’ and highlighted with red box. The number of TAL effectors in each strain is predicted if a full genome or high quality draft genome available, or by Southern blot analysis. The blank indicates that the number of TAL effector genes is unknown

Thirty-nine putative type III effectors (T3Es) genes were predicted from the genomic data, not including TAL effector genes, from diverse *X. translucens* strains (Fig. 5). Among them, twenty-three T3Es are conserved among the *X. translucens* pv. *undulosa*, *X. translucens* pv. *translucens*, and *X. translucens* pv. *cerealis*. The core set of T3Es includes AvrBs2, XopF, XopK, XopL, XopN, XopP, XopQ, XopR, XopX and XopZ, with the exception of two *X. translucens* pv. *cerealis* strains, which have frameshift mutation in XopR. Multiple copies of *avrBs2, xopF, xopX, xopL* and *xopP* are found in all three *X. translucens* pathovars, while strains from group 2 *Xanthomonas* species only harbor one.

Effector composition varies between different and within pathovars. The presence and absence, copy number difference, and frameshift in coding sequences of T3E genes were observed (Fig. 5). The gene *avrBs1* is present in only *X. translucens* pv. *undulosa* strain CS4 and both *X. translucens* pv. *cerealis* strains. The *xopJ1* effector gene is present in XT5523, XT-Rocky, XT5770, and DAR61454, but absent in XT4699, LG48, LW16, and XT5791. The gene *xopJ1* is present in all four *X. translucens* pv. *translucens* strains but not in either of *X. translucens* pv. *cerealis* strains. The *xopAH* and *xopAK* genes are present in all *X. translucens* pv. *undulosa* and *X. translucens* pv. *translucens* strains but not in either of *X. translucens* pv. *cerealis* strains. Five different *xopE* effector genes are present in the four pathovars. All *X. translucens* pv. *undulosa* strains have *xopE1* and *xopE5*, and *X. translucens* pv. *translucens* strains harbor *xopE2* and *xopE3*, while *X. translucens* pv. *cerealis* strains have *xopE1*, *xopE2* and *xopE5*. The *X. translucens* pv. *graminis* strain Xtg29 has *xopE1*, *xopE2* and *xopE4*. Besides the difference on the presence and absence of T3Es, variability in gene copy number exists among pathovars or within a pathovar. Two *xopAF* in *X. translucens* pv. *undulosa* strains DAR61454, XT-Rocky, XT5523, and XT4699, but only one copy in *X. translucens* pv. *undulosa* strains LW16, CS4, and XT5791. All four *X. translucens* pv. *translucens* strains have two copies of *xopAF,* while *X. translucens* pv. *cerealis* strains harbor one. Two copies of *xopL* are present in all *X. translucens* pv. *undulosa* and *X. translucens* pv. *translucens* strains, while there are four copies in *X. translucens* pv. *cerealis* strains. Two copies of *xopP* are present in *X. translucens* pv. *undulosa* strains, and three copies in *X. translucens* pv. *translucens* and *X. translucens* pv. *cerealis* strains with the exception that *X. translucens* pv. *translucens* strain B1 only has two *xopP* genes Four *X. translucens* pv. *undulosa* strains have frameshift mutations in one *xopP*. Consistent with a previous study on T3E genes in *X. axonopodis* pv. *manihotis* [21], frameshift mutations of T3Es is not rare in *X. translucens* strains. Frameshifts in the coding sequence of one *avrBs2* is present in *X. translucens* pv. *undulosa* strain P3. Two *X. translucens* pv. *cerealis* strains have frameshift mutations in *xopR* compared to *X. translucens* pv. *undulosa* and *X. translucens* pv. *translucens* strains. All four *X. translucens* pv. *translucens* strains harbor frameshift mutations in *xopAP* compared to *X. translucens* pv. *undulosa* and *X. translucens* pv. *cerealis* strains (Fig. 5).

### TAL effector gene content

Eight TAL effector genes were identified in XT4699. The number and lengths of TAL effector genes of XT4699 were confirmed by DNA Southern hybridization, in which the size of *Bam*HI fragments with TAL effector genes was consistent with the size of blotted bands (Additional file 11: Figure S7). Moreover, Sanger sequences of all eight TAL effector genes cloned by PCR with high fidelity polymerase were identical to the sequences of corresponding TAL genes in the assembly genome of XT4699 (Additional file 2: Table S2).

**Fig. 6:**
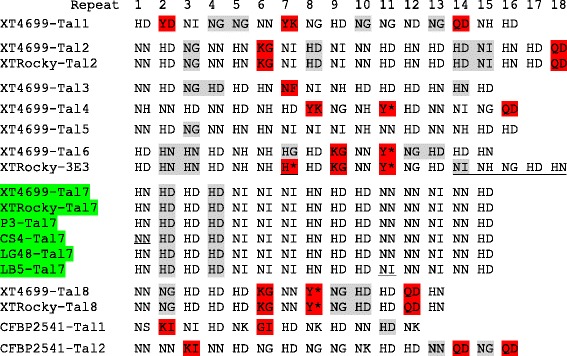
Alignment of RVDs of TAL effectors of *X. translucens* strains. The unusual RVDs are marked with red color. The RVDs of repeats with 34 amino acids are marked with gray color. The missing 13^th^ amino acid in the repeat is represented by *. The conserved TAL effector Tal7 is marked with green color. Variable RVDs among highly similar TAL effectors are underlined

**Fig. 7:**
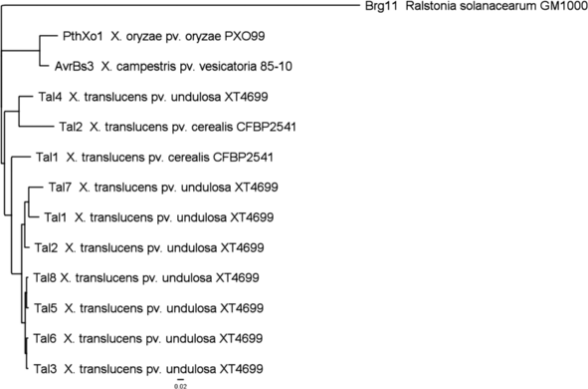
Phylogenetic tree of the concatenated N-terminal and C-terminal amino acid sequences of TAL effectors. The tree was constructed with the Tamura-Nei genetic distance model and the Neighbor-joining method, with Brg11 TAL effector from *Ralstonia solanacearum* GMI1000 as an outgroup using Geneious software. The scale bar with 0.02 indicates number of nucleotide substitutions per site

Four TAL effector genes from strain XT-Rocky were cloned and sequenced. Alignment of the predicted TAL effector proteins as represented by the repeat variable di-residues (RVDs) indicated that all the four of the TAL effectors that were retrieved from strain XT-Rocky were identical to corresponding TAL effectors from XT4699 except XTRocky-3E3 effector had variation in the last 5 RVDs and one RVD in the 7^th^ repeat compared to XT4699-Tal6 (Fig. 6). Two TAL effector genes from the *X. translucens* pv. *cerealis* strain CFBP2541 were previously identified [10]. TAL effector genes were not identified in the genome of *X. translucens* pv. *graminis* ART-Xtg29 strain [9]. The sequences of all RVDs available from these three strains reveal unusual RVDs, including YD, YK, QD, KG, Y*, NF, KI and GI, which are rarely or not found in *X. oryzae* and *X. campestris* strains of group 2 xanthomonads (Fig. 6, Additional file 12: Table S5). In addition, sequences of 32^nd^-35^th^ amino acids of repeats in TAL effectors in *X. translucens* are variable. Also, both 34 and 35 amino acids predicted repeats were observed within most individual TAL effectors (Additional file 12: Table S5). XT4699-Tal4 is distant from other TAL effectors in *X. translucens* based on the phylogenetic relationship of N- and C-terminal amino acid sequences (Fig. 7). TAL effectors in *X. translucens* strains are more closely to the AvrBs3 and PthXo1 of group 2 xanthomonads than Brg11 in *Ralstonia solanacearum* GMI1000 (Fig. 7, Additional file 13: Figure S8). The two TAL effectors from *X. translucens* pv. *cerealis* strain CFBP2541 are distinct from TAL effectors of *X. translucens* pv. *undulosa* strains in both RVD alignment and homology of N- and C-terminal amino acid (Figs. 6 and 7). The XT4699 genome annotation shows that most TAL effector genes are flanked by transposon elements (Additional file 14: Figure S9).

Primer pairs used for amplification of the TAL genes in XT4699 were applied for eleven other *X. translucens* pv. *undulosa* strains to identify potentially conserved TAL effector genes in *X. translucens* pv. *undulosa*. PCR fragments with similar sizes to *tal7* of XT4699 were present in 7 of 11 strains and sequencing of four representative genes showed the same RVDs as XT4699-Tal7 except for one amino acid variation in corresponding TAL effectors of both CS4 and LB5 (Fig. 6, Additional file 15: Figure S10).

### TAL effectors from XT4699 are associated with modulations in host gene expression

Microarray analyses of gene expression from wheat leaf samples that were inoculated with strains XT4699, XT-Rocky, and XT4699*hrcC*^−^ were performed to identify changes in host gene expression associated with pathogenicity. Gene expression profiles upon inoculation of wheat cultivar ‘Chinese Spring’ with strains XT4699 and XT4699*hrcC*^−^ mutant were found to be distinct from each other, revealing approximately 250 genes with at least a 10-fold difference of expression (Additional file 16: Table S6). Mutations were generated in TAL effector genes of XT4699 (Additional file 17: Figure S11), and analysis of host gene expression after inoculation of each TAL effector gene mutant revealed three differentially induced genes, corresponding to Affymetrix probes Ta.7291.1.S1_s_at, Ta.14824.1.S1_at and Ta.9765.1.S1_at, selected from the microarray analysis of XT4699 vs XT4699*hrcC*^−^ comparison (Additional file 16: Table S6), were not induced during infections of mutants M2 (*tal6*^*−*^), M3 (*tal7*^*−*^) and M4 (*tal1*^*−*^), respectively (Additional file 18: Figure S12). The probes sets Ta.7291.1.S1_s_at, Ta.14824.1.S1_at and Ta.9765.1.S1_at corresponded to coding regions that were predicted to encode a succinate dehydrogenase subunit, a choline transporter related protein and a cell wall invertase, respectively.

**Fig. 8:**
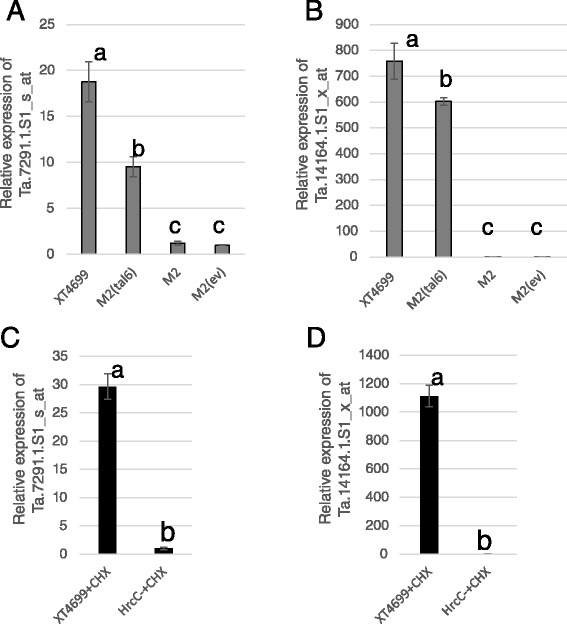
XT4699-Tal6 effector modulates gene expression in wheat. First leaves of 10-day-old Chinese Spring wheat plants were inoculated with XT4699, M2, M2 (empty vector, ev for short) and M2 (*tal6*), respectively. Inoculated leaves were collected 24 h post inoculation. Gene specific primers were used for qRT-PCR in each panel. Relative expressions of Ta.7291.1.S1_s_at (**a** and **c**) and Ta.14164.1.S1_s_at (**b** and **d**) were calculated; In (**a**) and (**b**), the fold change relative to M2 (ev) treatment was calculated with 2^-∆∆Ct^ method. In (**c**) and (**d**), bacterial strains treated with 100 μm cycloheximide (CHX) were applied for inoculation. The fold change relative to *hrcC* ^−^ + CHX treatment was calculated with 2^-∆∆Ct^ method. The lowercase letters indicate significantly different groups with *P*-value < 0.05 in the ANOVA statistics analysis. Two biological replicates were applied, and the experiments were repeated twice with consistent results

Two genes from the microarray analysis, corresponding with to probes Ta.7291.1.S1_s_at and Ta.14164.1.S1_x_at, were induced by infection with wild type XT4699 but not by strain XT-Rocky (Additional file 16: Table S6). M2, a mutant of *tal6*, failed to induce Ta.7291.1.S1_s_at (Fig. 8a, Additional file 18: Figure S12) and Ta.14164.1.S1_x_at, which is predicted to encode a bHLH family transcriptional factor (Fig. 8b). Comparison of RVDs of TAL effectors between XT4699 and XT-Rocky revealed one TAL effector, XTRocky-3E3, is highly similar to XT4699-Tal6 but has variations in 6 RVDs (Fig. 6). The qRT-PCR assays indicated these two genes were not induced by *X. translucens* pv. *translucens* XT8, *X. translucens* pv. *cerealis* XT123 or *X. translucens* pv. *undulosa* XT5523 (the latter being very close to XT-Rocky in phylogeny). The two genes were elevated in expression after infection of plants by three other *X. translucens* pv. *undulosa* strains (Additional file 19: Figure S13). Complementation of the M2 mutant with *tal6* resulted in restored expression of both genes (Fig. 8a and b). The transcriptional induction of the two genes by XT4699 occurred in the presence of the eukaryotic translation inhibitor cycloheximide (CHX) (Fig. 8c and d), which had been used for distinguishing direct or indirect target of TAL effectors from *Xanthomonas* strains in previous study [22, 23].

## Discussion

A complete genome assembly provides an excellent foundation for further genetic and comparative analyses into the pathogenicity mechanisms of a pathogen. The advances of genome sequencing technologies and better assembly algorithms now facilitate assembly of genomes with highly repetitive regions [24]. In this study, a complete genome sequence was generated for *X. translucens* pv. *undulosa* strain XT4699. The results demonstrated that PacBio data was sufficient to achieve a high-accuracy assembly of a bacterial genome with many IS elements and multiple copies of highly repetitive TAL effector genes. Intact sequences of TAL effector genes can be used to predict the potential targets in host genomes, thereby facilitating the identification of possible resistant or susceptible genes [25, 26]. Draft genome sequence data was also generated using Illumina short reads for 20 strains, including XT4699, to determine the relatedness and compare effector contents among them. The comparison between the XT4699 Illumina-only assembly and the complete XT4699 assembly shows that approximately 97 % the complete genome is covered by the Illumina-only assembled contigs. The characterization of assembly gaps on the Illumina-only assembly indicates that gaps are largely located at repetitive regions and high GC contents. The lengths of gaps at repetitive regions tend to be linearly correlated to repeat lengths while the gaps at high GC regions are typically small. Gaps due to a high GC content may, in the future, be reduced by using a new Illumina PCR-free library preparation protocol, which is expected to ameliorate amplification biases associated with high GC contents. TAL effector gene content was not resolved in the Illumina draft genome sequences. To resolve repeats, especially long and complex repeats, PacBio or other emerging long read sequencing technologies are needed [27, 28]. The improvement of these long read sequencing technologies may dramatically reduce the sequencing cost per bacterial strain in the near future. The ability to sequence and completely assemble a large number of independent strains would greatly accelerate genome comparison and the identification of bacterial virulence factors. Although not examined here, PacBio sequence can also be used to characterize the methylomes of bacteria and enables the exploration of possible epigenetic features in bacterial gene expression and restriction modifications [29].

The comparison of type III effector (T3E) content in *X. tranlucens* strains identified both conservative (*N* = 23) and variable T3Es among three pathvors, *X. translucens* pv. *undulosa, X. translucens* pv. *translucens* and *X. translucens* pv. *cerealis*. Effector triggered immunity (ETI) is defined by T3S/R gene pairs, which are present in the pathogens and host plants, respectively [30, 31]. Highly conserved sets of T3Es provide a potential opportunity to deploy pyramided R genes for broad resistance to the bacterial leaf streak disease, although no T3Es-specific R genes are known in wheat at this time. Presence and absence variation, copy number variation, and frameshift mutations were all observed in T3Es among *X. translucens* strains, and TAL effector repertories are also highly variable. The *X. translucens* pv. *cerealis* strain CFBP2541 only contains two TAL effectors, sharing no similarity with TAL effectors from *X. translucens* pv. *undulosa* strains based on the RVD sequences. Variation in T3Es may be one factor accounting for pathogenic diversity and host range determinants of *X. translucens* strains. Pathogenic and genetic diversity are present in *X. translucens* strains collected from five different locations in North Dakota [2], and host specific virulence genes have been reported to act as host range determinants [32, 33]. CFBP2541 (*X. translucens* pv. *cerealis*) is a phylogenetically close strain to XT123 (*X. translucens* pv. *cerealis*) and was reported to cause severe symptoms on ‘Morex’ barley [10]. XT123 only induced chlorosis on ‘Morex’ barley. However, pathogenicity diversity among different *X. translucens* strains may also be derived from variation in other genetic elements related with bacterial fitness. Functional mutation and complementation in genes related with virulence is needed for detecting the virulence contribution of genes and explaining their roles leading to difference in pathogenicity types.

The TAL effector triggered susceptibility has been demonstrated in several disease complexes. The TAL effectors are a large family of closely related type III effector proteins, which transcriptionally activate host gene expression by directly interacting with promoter elements of host genes and have a varying degree of contribution in bacterial virulence, proliferation, and other disease symptomatology [34, 35]. So far, TAL effectors with virulence contribution have been detected in strains of *X. oryzae* pv. *oryzae*, *X. citri* pv. *citri*, *X. campestris* pv. *vesicatoria*, *X. campestris* pv. *malvacearum*, *X. axonopodis* pv. *manihotis* and *X. oryzae* pv. *oryzicola* [22, 36–44]. However, it is still an open question whether TAL effectors play roles in triggering susceptibility in bacterial leaf streak on wheat. In this study, we are confirmed that TAL effectors do modulate transcriptional profiling of wheat host. The XT4699-Tal6 effector affects the transcriptional level of two candidate target genes, encoding a bHLH transcriptional factor and a succinate dehydrogenase subunit, respectively. Whether or not they are directly targeted by Tal6 is unknown. Searching in the promoter regions of the two genes using the RVD sequence of Tal6 did not give positive EBE above the default cutoff value (https://tale-nt.cac.cornell.edu/node/add/talef-off). We also failed to detect the direct interaction between Tal6 and 400 bp promoter sequence upstream of translation start site of bHLH gene in the assays of *Agrobacteria*-mediated transient expression in *Nicotiana tabacum* KY-14 (data not shown). In this study, no *tal* mutants suffered an obvious loss of virulence when compared to the WT strain in the bacterial growth population assays (Additional file 10: Figure S6). It is not known if TAL effectors contribute to bacterial virulence in other ways. Previous studies showed AvrBs3 of *X. campestris* pv. *vesicatoria*, Tal2g of *X. oryzae* pv. *oryzicola* and Avrb6 from *X. campestris* pv. *malvacearum* did not affect bacterial growth but had roles in other types of virulence contributions [37, 38, 42].

In this study, XT4699-*tal7* was identified as a conserved TAL effector gene among 8 of 12 *X. translucens* pv. *undulosa* strains. Cloning and sequencing of TAL effector genes in larger collection of *X. translucens* strains is needed to get more reliable information regarding with conservation of TAL effector genes. The insertion of DNA elements, specifically designed to be recognized by RVDs of conserved TAL effectors, in the promoter regions of effective R genes was applied for controlling bacterial blight and bacterial leaf streak of rice [45, 46]. Therefore, for controlling the bacterial leaf streak on cereal crops, use of the RVDs of conserved *X. translucens* TAL effectors may allow the engineering of specific super terminator R genes in cereal crops.

## Conclusions

Complete genome assembly of repeat laden bacterial genomes can be facilitated by long read sequencing platforms. Genomic comparisons among strains from different pathovars of *X. translucens* or within one pathovar of *X. translucens* revealed variations in type III effector repertories, which may explain the pathogenic and genetic diversity of *X. translucens* strains and might relate with host range adaptations of specific strains [32, 33]. TAL effectors in *X. translucens* strains are distant from ones of group 2 xanthomonads in phylogeny and RVD sequences. Gene expression studies reveal specific TAL effectors in XT4699 function in modulating expression of specific genes in wheat plants.

## Methods

### Bacterial strains and genomic DNA extraction

The *X. translucens* strains used in this study are listed in Table 2. *Xanthomonas* strains were grown on tryptone sucrose agar (tryptone, 10 g/l; sucrose, 10 g/l; glutamic acid, 1 g/l; Difco Bacto agar, 15 g/l) medium at 28 °C [47]. Bacterial strains were stored in nutrient broth with 20 % glycerol at −80 °C. For genomic DNA extraction, fresh grown bacteria from medium was washed first by sterilized water and be treated with 1 % SDS and proteinase K (1 mg/ml) at 37 °C for 20–30 min. Samples were then treated with 0.5 M NaCl and incubated at 65 °C for 30 min for cell lysis. Phenol and chloroform extraction and 2.5 times 95 % ethanol precipitation were applied to isolate DNA. Samples were treated with RNase A (0.1 mg/ml) and incubated at 37 °C for 30 min for RNA removal.

### Genome sequencing and assembly

Bacterial genomic DNAs were subjected to library preparation using the Illumina TruSeq DNA LT Sample Prep kit. 2x250 bp paired-end data for all sequenced strains were generated on an Illumina MiSeq at the Integrated Genomic Facility at Kansas State University. Reads were subjected to adaptor and quality trimming and assembled *via* CLC Genomics Workbench software, DISCOVAR *de novo* (ftp://ftp.broadinstitute.org/pub/crd/DiscovarDeNovo/), or SOAPdenov2 [48]. For the whole genome assembly for XT4699 strain, the 10-kb libraries were constructed, quantitated, and sequenced on the two SMRT cells of PacBio RS II at the Interdisciplinary Center for Biotechnology Research (ICBR) at University of Florida. These reads were assembled into three contigs using an optimized PacBio pipeline, HGAP2 [12]. As suggested by a PacBio HGAP instruction for bacterial assemblies, the parameter of “Target Coverage” was changed from 30 to 15. One short contig was discarded due to low coverage of PacBio reads. Two remaining contigs were merged into a single contig with Illumina assembled contigs using the minimus2 module in AMOS (sourceforge.net/apps/mediawiki/amos). The contig was further circularized through removing the overlap at two ends. To improve the quality of the draft assembly at the ends of the contig, a standard PacBio resequencing pipeline with PacBio reads was used for additional error correction.

### Alignment of XT4699 Illumina reads to the assembled genome to assess the assembly quality

Trimmed Illumina reads were aligned to the assembly sequences with BWA-MEM [49]. The alignment was subjected to stringent filtering criteria (minimum mapping score: 40; minimum overlap: 100 bp; minimum identity: 97; and minimum read coverage: 98 %) to obtain a set of uniquely and confidently mapped reads coverage. To identify mismatches between Illumina sequencing data and the assembly genome, GATK was applied to discover SNPs and INDELs [50, 51]. A set of criteria for polymorphism filtering, including a minimum polymorphic site coverage set as five reads and minimum percentage of reads of the polymorphic allele set at 90 %, were used as the filter to identify the mismatches between Illumina sequences and the assembled genome.

### Genome annotation

The finished assembled genome of XT4699 was annotated using the NCBI Prokaryotic Genome Automatic Annotation Pipeline (PGAAP) (ncbi.nlm.nih.gov/genome/annotation_prok). The annotated genome has been deposited in Genbank with accession number (CP_008714). The complete and draft genomic sequences were also annotated using the RAST (Rapid Annotation using Subsystem Technology) (http://rast.nmpdr.org) [52]. The sequence feature of TTCGN16TTCGN, where N represents A, T, G, and C, was used to search the 500-bp promoter region before each annotated gene to identify genes with the plant inducible promoter (PIP) element. IS elements of XT4699 genome was annotated by ISfinder (www-is.biotoul.fr) [53].

### Phylogenetic tree construction

Multilocus sequence analysis (MLSA) was used to build phylogenetic trees in *Xanthomonas* strains [54]. To generate the phylogenetic relationship tree, concatenated sequences of four conserved loci (*dnaK*, *gyrB*, *groEL* and *recA*) were assembled. The distance matrix was constructed with the Tamura-Nei genetic distance model and the tree was built using the Neighbor-joining method in the Geneious software R6, with sequence of *Stenotrophomonas maltophilia* K279a as outgroup.

Whole-genome discovery of single nucleotide polymorphisms (SNPs) was performed by using the assembly draft or finished assembly sequences with MUMmer 3.0 [14]. The modules of nucmer, delta-filter, and show-snps were sequentially run to identify SNPs. At least 100 bp and 90 % identity of one-to-one match was required for the alignment between each assembled sequence and the XT4699 reference genome. After identifying all the SNPs of each strain, the XT4699 reference genome was modified at all the SNP sites to polymorphic bases. The resulting modified XT4699 sequence was used as the reference for the second run of the SNP discovery. Consequently, a matrix of the genotyping result of all the strains was obtained. The genotyping data at the SNP sites with no missing data were used to construct a phylogenetic tree using an R package APE [55].

### Identification of type III effector genes

A combined resource of Type III effector (T3E) in the genus of *Xanthomonas*, also called Xop effectors, was used as blastp query against the NCBI and RAST annotation protein database of XT4699 [1, 8, 9, 56]. The cutoff e-value was set as 1e-10. For other *X. translucens* strains, the same query was applied using blastp against the RAST annotation database of each strain. In the second round of identification of T3Es, the same query was applied against genome sequence of each strains using tblastn to identify T3Es, which may be overlooked by annotation process. This tblastn search was also used to confirm the presence, absence and number of copies and frameshift mutation of T3Es in the genome. Assembled contigs with less than 5x sequencing coverage, which might contain relatively high sequence errors, were excluded. Frameshifts in T3Es were identified when multiple reading frames from different segments of a contig were matched to the separated regions of a protein. Premature termination codons were identified when translation was truncated. The missing proteins overlooked by the annotation but identified by tblastn were re-evaluated by performing blastp in the NCBI protein database.

### Southern blot analysis

Genomic DNA manipulation was performed according to standard protocols [57]. Genomic DNA of *X. translucens* strains was completely digested by *Bam*HI (New England Biolabs, MA). The digested DNA was separated in 0.9 % agarose gel *via* electrophoresis at 4 °C at 40 Volt overnight. The *sphI* fragment of TAL effector gene in *X. oryzae* pv. *oryzae* strain AXO1947 was used as the probe. AlkPhos Direct Labeling and Detection System with CDP-Star Kit (GE healthcare) was applied for the probe labeling, hybridization and detection procedures.

### Mutagenesis and mutant validation in XT4699

A *hrcC*^*−*^ mutant of XT4699 was generated by gene transfer and homologous recombination of a mutant copy. The partial fragment of *hrcC* gene were amplified by PCR with primers XTThrcC-F and XTThrcC-R (Additional file 20: Table S7) and cloned into suicide vector pKNOCK-Km vector [58]. The suicide vector with cloned fragment was transformed into *E. Coli* S17–1 pir strain for bacterial conjugation. The mixture of XT4699 and S17–1 strains was plated on NA at 28 °C for 24 h, then transferred to TSA plates containing 20 ug/ml Cephalexin and 50 ug/ml kanamycin for selection of XT4699*hrcC*^*−*^ mutants [59]. The mutants were validated by PCR with primers XTThrcC-Out and 07KM-Val. The TAL mutants of XT4699 were generated in similar way. Partial fragment of N-terminal region of each TAL effector gene in XT4699 was individually amplified by PCR with primers 4699 N-TAL-F and 4699 N-TAL-R. The partial fragment was cloned into pKNOCK-Km vector for conjugation. Mutants were validated by PCR with specific primers located upstream of each TAL gene and the reverse primer Forall-Val in the vector (Additional file 20: Table S7).

### Pathogenicity assays

Two-day old *Xanthomonas* cultures were scraped off the TSA plates and resuspended in sterilized water. For pathogenicity type assays, bacterial suspensions (OD600 = 0.2) were infiltrated onto same age leaves of wheat and barley plants by needleless syringe. Hexaploid wheat cultivars ‘Chinese Spring’, ‘Jagger’, ‘Hope’ and ‘Canthatch’, one *Triticum turgidum* wheat (accession number 107 in WGRC at KSU), ‘KS Southeast’ and ‘Morex’ barleys were applied. The symptoms were observed and photographed at 4DPI. For dip inoculation assays, second leaves of 14-day-old ‘Chinese Spring’ wheat plants were dipped into the bacterial suspensions (OD600 = 0.2) coated with 0.02 % Silwet L–77. The water soaking symptoms appeared at 4–5DPI and were photographed at 8DPI. For bacterial growth population assays, equal amount of inoculum of 4 × 10^4^ dilutions of original bacterial suspension (OD600 = 0.25) was infiltrated on 2^nd^ leaves of 3-week-old ‘Chinese Spring’ wheat plants by needleless syringe. At 6DPI, three biological replicates of inoculated leaves (3 cm) were pooled and ground together. Ground samples were serially diluted, and 100 μl of diluted samples were added onto TSA agar plates for colony counting three days later. 10–100 colonies per plate is considered as optimal and three plates were applied for calculating colony forming units (CFU).

### Microarray and qRT-PCR

Fully expanded leaves of 10-day-old ‘Chinese Spring’ wheat plants were inoculated with three different bacterial suspensions (XT4699, XT-Rocky and XT4699*hrcC*^*−*^), each at an optical density of 0.5 at 600 nm, using a needleless syringe. RNA samples were isolated 24 h after inoculation using Trizol reagent (Invitrogen) as described by the manufacturer. Three biological replicates, each of which was pooled from three independently inoculated leaves, were used for each treatment. RNA quality was assessed using an Agilent 2100 Bioanalyzer (Agilent Technologies). Processed samples were hybridized to GeneChip Wheat Genome Array (Affymetrix) at Integrated Genomics Facility at Kansas State University. MAS5 algorithm normalization was applied using Affymetrix default parameters. Microarray analyses were performed with the Bioconductor/R package Limma [60]. Linear models and empirical Bayes methods were used for statistical tests of differential expression [61]. The comparisons were as follows: XT4699 vs XT4699*hrcC*^*−*^ and XT-Rocky vs XT4699*hrcC*^*−*^. The *P*-values of all probes were subjected to multiple tests correction to estimate false discovery rate (FDR) [62]. Probes were considered differentially expressed if they had an adjusted *P*-value <0.05 and a fold-change greater than two. Raw and processed data are accessible from Gene Expression Omnibus (http://www.ncbi.nlm.nih.gov/geo) with accession number GSE73757. All probes were annotated using Blastx analysis (E-value < e-30) against the GenBank nonredundant protein database. A few probes of interest, which show low *p*-values, were manually annotated. For qRT-PCR, 1 μg RNA samples were treated with DNaseI following the protocols provided by Invitrogen and subjected to reverse transcription reaction using iScript cDNA synthesis kits (Bio-rad). 10x diluted samples of the cDNA products were applied for real-time PCR in the CFX-96 machines at the Integrated Genomics Facility at Kansas State University. Relative gene expression level was calculated with 2^-∆∆Ct^ method [63]. The EF-1α gene was set as an internal control gene. Primer sequences are shown in the Additional file 20: Table S7.

### Availability of supporting data

The complete genome sequence of *X. translucens* pv*. undulosa* XT4699 and its annotation are accessible in Genbank under the accession number CP_008714. The raw PacBio and Illumina reads of XT4699 were also submitted to Genbank Sequence Read Archive (SRA) under the BioProject number of PRJNA248137. The datasets of raw Illumina sequencing reads and draft genome assembly sequences of nineteen additional *X. translucens* strains were submitted to GenBank under the BioProject number of PRJNA302486. The raw and processed microarray data are available from Gene Expression Omnibus with accession number GSE73757. Additional files 1, 2, 3, 6, 12, 16, 20: Tables S1-S7 and Additional files 4, 5, 7, 8, 9, 10, 11, 13, 14, 15, 17, 18, 19: Figures S1-S13 are included in Additional files.

## Additional files

Additional file 1: Table S1. The sequence difference between the reference genome and the Illumina assembly for XT4699. (XLSX 9 kb) Additional file 2: Table S2. Summary of alignments of Sanger reads from TAL effector genes to the assembly genome of XT4699. (XLSX 10 kb) Additional file 3: Table S3. Genes with plant inducible promoter (PIP) box in XT4699. (XLSX 14 kb) Additional file 4: Figure S1. Data coverage of XT4699 PacBio and Illumina sequences. A) Coverage of PacBio sequences (light blue) and Illumina sequences (orange) across the genome. B) The relationship between Illumina sequencing coverage and GC% of non-overlapping 200 bp windows. C) Visualization of the alignment of Illumina assembled contigs on the XT4699 reference genome. D) The relationship between PacBio sequencing coverage and GC% of non-overlapping 200 bp windows. (PDF 487 kb) Additional file 5: Figure S2. The relationship between genome repeats and assembled gaps using XT4699 Illumina data. A) The repeat features (length, similarity between repeated copies, and copy number) of assembled repeats and unassembled repeats. B) The relationship between assembled gaps and repeat lengths. (PDF 247 kb) Additional file 6: Table S4. SNP difference count among *Xanthomonas* strains in a total of 9836 SNPs. (XLSX 13 kb) Additional file 7: Figure S3. Different disease symptoms induced by pathovars of *X. translucens*. In A-G panels, different wheat and barley cultivars were inoculated. A, ‘Chinese Spring’ wheat; B, ‘KS Southeast’ barley; C, ‘Morex’ barley; D, ‘Jagger’ wheat; E, ‘Hope’ wheat; F, ‘Canthatch’ wheat; G, Triticum turgidum wheat #107. In A, B, C, plants are 3 weeks old and second leaves were inoculated. In D, E, F, G, leaves with similar age from 50-day-old plants were inoculated. (PDF 359 kb) Additional file 8: Figure S4. Phylogenetic tree based on CRISPR *Cas* loci of *X. translucens* strains. The sequence of CRISPR *Cas* genes is annotated in RAST website http://rast.nmpdr.org. The phylogenetic tree was generated using Geneious software Version 6 with the Tamura-Nei genetic distance model and the Neighbor-joining method, with PXO99 CRISPR *Cas* genes as outgroup. The scale bar indicates number of nucleotide substitutions per site. (PDF 149 kb) Additional file 9: Figure S5. Syntenic gene clusters of type III secretion system among *X. translucens* strains. XT-Rocky and XT4699 are *X. translucens* pv. *undulosa* while ART-Xtg29 is *X. translucens* pv. *gramins*. White arrows (drawn to scale) represent orientation and position of Hrp genes. Black arrows indicate the two predicted IS elements in ART-Xtg29 strain. The names of Hrp genes are shown above or under the white arrows. The identity of homologous proteins of compared strains is shown between them. (PDF 155 kb) Additional file 10: Figure S6. Bacterial population assay. Equal amount of 4x10^4^ dilution of bacterial inoculum of different *tal* mutants, *hrcC* mutant and WT were inoculated on 3-week-old Chinese Spring wheat by needleless syringe. At 6DPI, three inoculated leaves (3 cm) were pooled and ground together in each treatment. Samples were serially diluted and 100 μl of diluted samples were added to TSA plates. Plates were incubated at 28 °C for colony formation. Data represents the mean of Log10 CFU/cm leaf ± standard deviation. The * indicates the significant difference of bacterial population of *hrcC* ^*−*^ mutant compared to other *tal* mutants or WT at 6DPI under *p*-value <0.001 in the ANOVA statistics analysis. (PDF 157 kb) Additional file 11: Figure S7. Southern blot analysis of two *X. translucens* pv. *undulosa* strains from Kansas. Genomic DNA digested by *Bam*HI and hybridized by *sph*I fragment of TAL effector gene in *X. oryzae* pv. *oryzae* strain AXO1947. The size of blotted bands is consistent with assembly genome of XT4699. (PDF 182 kb) Additional file 12: Table S5. Amino acid sequences of TAL effector proteins in *X. translucens* pv. *undulosa* strain XT4699. (PDF 135 kb) Additional file 13: Figure S8. Alignment of concatenated N-terminal and C-terminal amino acid sequences of TAL effectors. The sequences of Tal2 and Tal4 of *X. translucens* XT4699, PthXo1 of *X. oryzae* PXO99, AvrBs3 of *X. campestris* 85–10 and Brg11 of *Ralstonia Solanacearum* GMI1000 are applied for alignment, which is generated by Geneious software. (JPG 737 kb) Additional file 14: Figure S9. Most TAL effector genes are flanked by transposon elements. The blue arrows indicate the TAL effector genes and the dark red arrows show the transposase genes; the gray arrows indicate other genes. The genes are listed as following: 1, *RpoD*, encoding RNA polymerase subunit sigma-70; 2, Hypothetical genes; 3, Acetyltransferase and hypothetical genes; 4, Type III effector gene *xopL*; 5, *vgrG*, encoding type IV secretion protein. (PDF 168 kb) Additional file 15: Figure S10. XT4699-*tal7* is a conserved TAL effector gene. Primer pair used for cloning *tal7* gene in XT4699 was applied in PCR with template DNA of other strains from North Dakota and Kansas. PCR bands with similar size as XT4699-*tal7* were shown in the blue box. (PDF 118 kb) Additional file 16: Table S6. Microarray analysis of gene expression from inoculated wheat leaves by XT4699, XT-Rocky and XT4699*hrcC* ^−^. (XLSX 153 kb) Additional file 17: Figure S11. Validation of all eight TAL mutants in XT4699 by PCR. The sequence of specific primers for each TAL gene and the primer from the vector are provided in Table S6. The desired PCR product size is around 1.5 kb. The corresponding names of TAL genes are shown for all 8 mutants. A, M1 (*tal2* mutant), M2 (*tal6* mutant); B, M3 (*tal7* mutant), M4 (*tal1* mutant); C, M5 (*tal5* mutant), M6 (*tal8* mutant); D, M7 (*tal3* mutant), M8 (*tal4* mutant). (PDF 351 kb) Additional file 18: Figure S12. Induction of host genes is impaired by mutation of specific TAL genes. M2 is lack of *tal6*, M3 is lack of *tal7* and M4 is the mutant of *tal1*. A, relative expression level of Ta.7291.1.S1_at, corresponding to succinate dehydrogenase subunit gene, was calculated compared to M2 treatment; B, relative expression level of Ta.14824.1.S1_at, corresponding to choline transporter related gene, was calculated compared to M3 treatment; C, relative expression level of Ta.9765.1.S1_at, corresponding to cell wall invertase gene, was calculated compared to M4 treatment. 2^-∆∆Ct^ method was applied in calculation. The * indicates significant difference compared to other TAL mutants treated samples with *P*-value < 0.05 in the ANOVA statistics analysis. (PDF 191 kb) Additional file 19: Figure S13. Induction of Ta.14164.1.S1_s_at and Ta.7291.1.S1_s_at is strain specific. XT8 is *X. translucens* pv. *translucens*, XT123 is *X. translucens* pv. *cerealis*, XT130, XT5523, XT5770 and XT5791 are strains of *X. translucens* pv. *undulosa*. M2 is the mutant of XT4699 lack of *tal6*. The relative expression level is calculated relative to M2 treatment by with 2^-∆∆Ct^ method. A, the relative expression of Ta.14164.1.S1_s_at, corresponding to *bHLH* gene, was calculated; B, the relative expression of Ta.7291.1.S1_s_at, corresponding to succinate dehydrogenase gene, was calculated. The lowercase letters indicate significantly different groups with *P*-value < 0.05 in the ANOVA statistics analysis. (PDF 178 kb) Additional file 20: Table S7. Primers used in this study. (XLSX 8 kb)

## Abbreviations

BLS: Bacterial leaf streak
Cas: CRISPR-associated genes
CDS: Coding DNA sequence
CHX: Cycloheximide
CRISPR: Clustered regularly interspaced short palindromic repeat
DPI: Days post infection
HR: Hypersensitive reaction
INDELs: Insertion-and-deletions
IS: Insertion Sequence
MLSA: Multilocus sequence analysis
NRPS: Non-ribosomal peptide synthesis
PacBio: Pacific Bioscience
PGAAP: Prokaryotic Genome Automatic Annotation Pipeline
PIP: Plant inducible promoter
RVDs: Repeat variable di-residues
SMRT: Single molecule, real time
SNPs: Single nucleotide polymorphisms
T3Es: Type III effectors
T3SS: Type III secretion system
TAL: Transcription-Activator Like
