# Supplementary material for: Long read and single molecule DNA sequencing simplifies genome assembly and TAL effector gene analysis of Xanthomonas translucens
Source: BMC Genomics. 2016 Jan 5;17:21. doi: 10.1186/s12864-015-2348-9 (PMC4700564; doi:10.1186/s12864-015-2348-9)
Supplement: Additional file 4: Figure S1. — Data coverage of XT4699 PacBio and Illumina sequences. A) Coverage of PacBio sequences (light blue) and Illumina sequences (orange) across the genome. B) The relationship between Illumina sequencing coverage and GC% of non-overlapping 200 bp windows. C) Visualization of the alignment of Illumina assembled contigs on the XT4699 reference genome. D) The relationship between PacBio sequencing coverage and GC% of non-overlapping 200 bp windows. (PDF 487 kb) [file 12864_2015_2348_MOESM4_ESM.pdf]

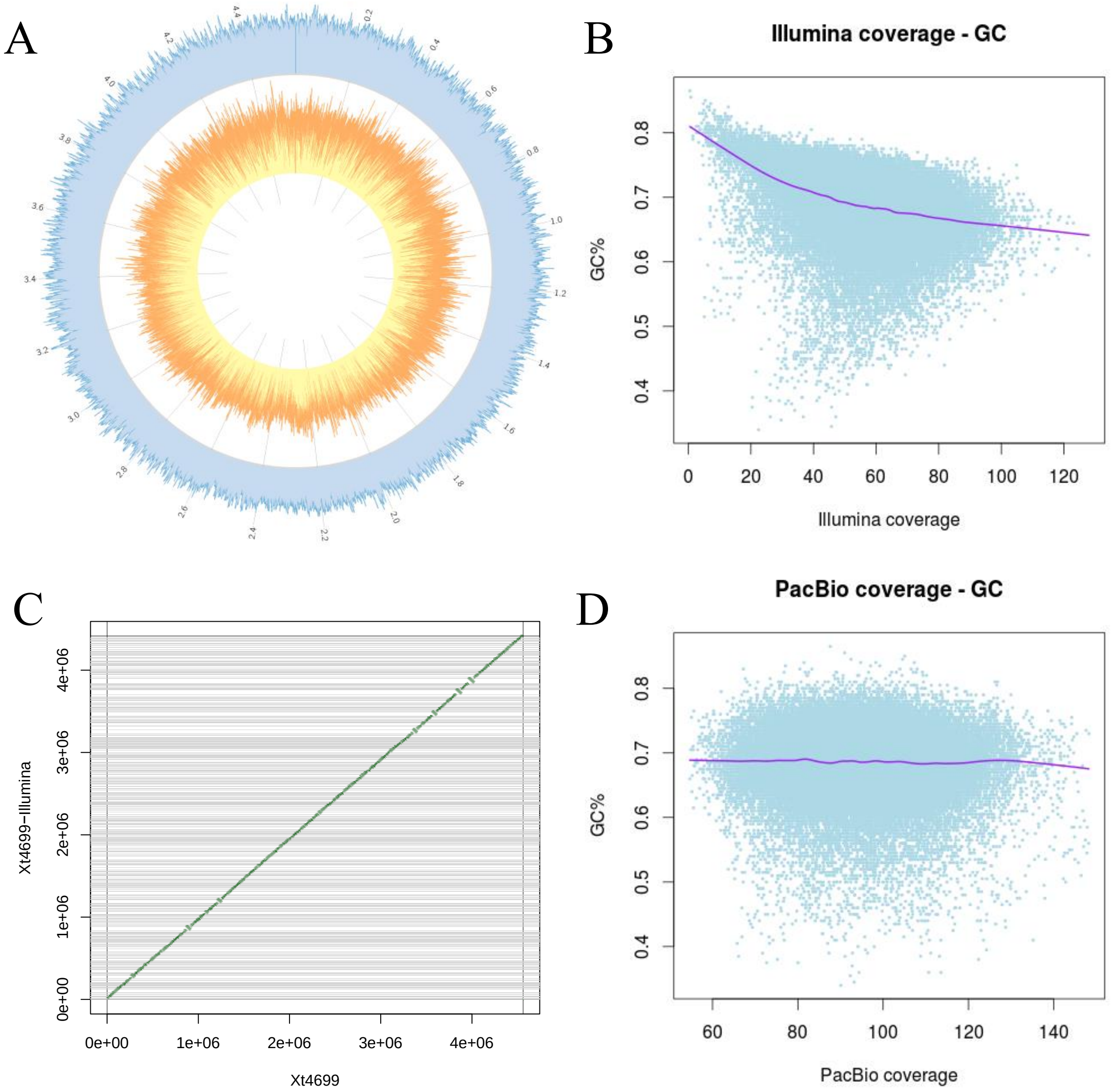

Figure S1. Data coverage of XT4699 PacBio and Illumina sequences. A) Coverage of PacBio sequences (light blue) and Illumina sequences (orange) across the genome. B) The relationship between Illumina sequencing coverage and GC% of non-overlapping 200 bp windows. C) Visualization of the alignment of Illumina assembled contigs on the XT4699 reference genome. D) The relationship between PacBio sequencing coverage and GC% of non-overlapping 200 bp windows.
