# Supplementary material for: Long read and single molecule DNA sequencing simplifies genome assembly and TAL effector gene analysis of Xanthomonas translucens
Source: BMC Genomics. 2016 Jan 5;17:21. doi: 10.1186/s12864-015-2348-9 (PMC4700564; doi:10.1186/s12864-015-2348-9)
Supplement: Additional file 5: Figure S2. — The relationship between genome repeats and assembled gaps using XT4699 Illumina data. A) The repeat features (length, similarity between repeated copies, and copy number) of assembled repeats and unassembled repeats. B) The relationship between assembled gaps and repeat lengths. (PDF 247 kb) [file 12864_2015_2348_MOESM5_ESM.pdf]

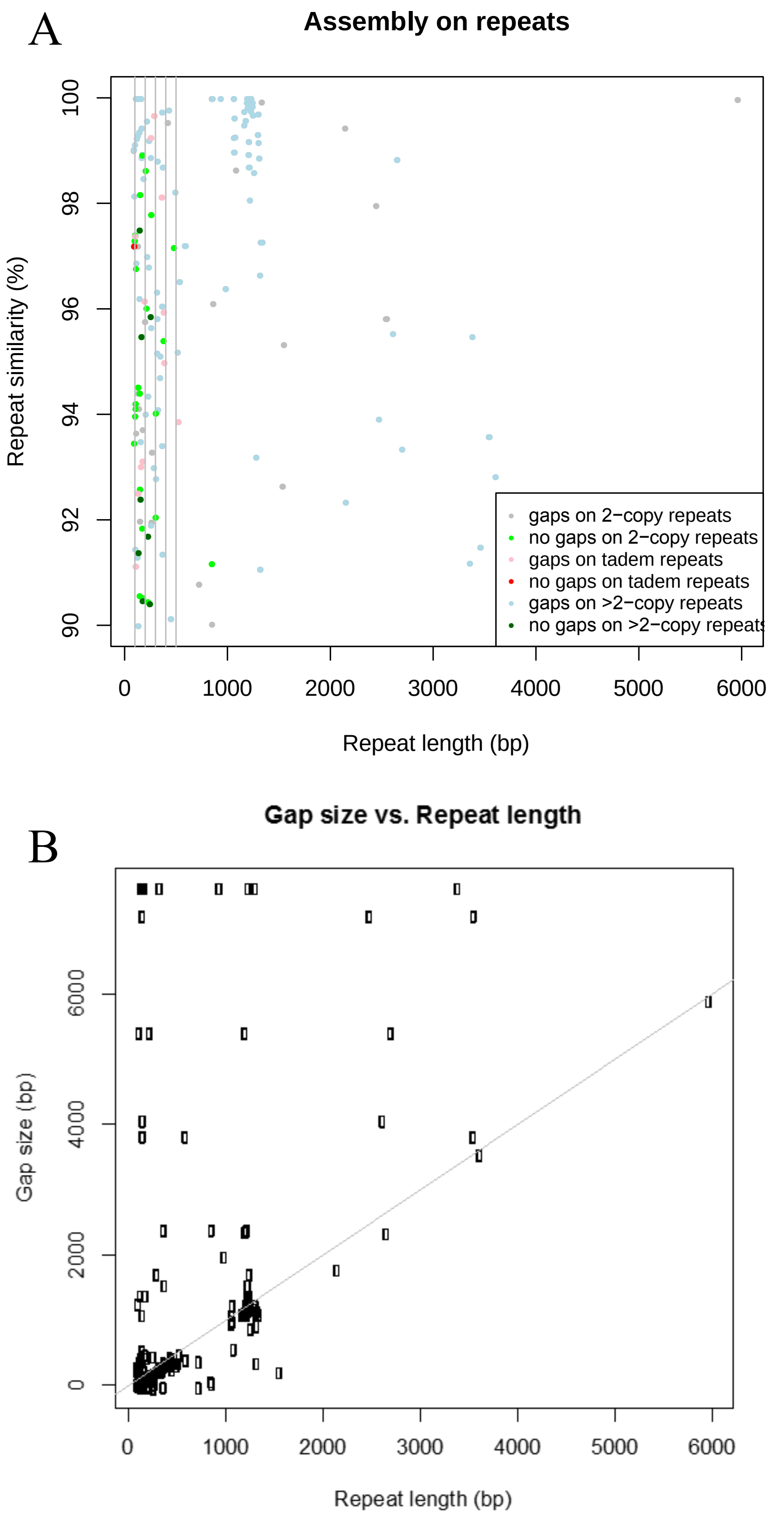

Figure S2. The relationship between genome repeats and assembled gaps using XT4699 Illumina data. A) The repeat features (length, similarity between repeated copies, and copy number) of assembled repeats and unassembled repeats. B) The relationship between assembled gaps and repeat lengths.
