# Supplementary material for: Long read and single molecule DNA sequencing simplifies genome assembly and TAL effector gene analysis of Xanthomonas translucens
Source: BMC Genomics. 2016 Jan 5;17:21. doi: 10.1186/s12864-015-2348-9 (PMC4700564; doi:10.1186/s12864-015-2348-9)
Supplement: Additional file 7: Figure S3. — Different disease symptoms induced by pathovars of X. translucens. In A-G panels, different wheat and barley cultivars were inoculated. A, ‘Chinese Spring’ wheat; B, ‘KS Southeast’ barley; C, ‘Morex’ barley; D, ‘Jagger’ wheat; E, ‘Hope’ wheat; F, ‘Canthatch’ wheat; G, Triticum turgidum wheat #107. In A, B, C, plants are 3 weeks old and second leaves were inoculated. In D, E, F, G, leaves with similar age from 50-day-old plants were inoculated. (PDF 359 kb) [file 12864_2015_2348_MOESM7_ESM.pdf]

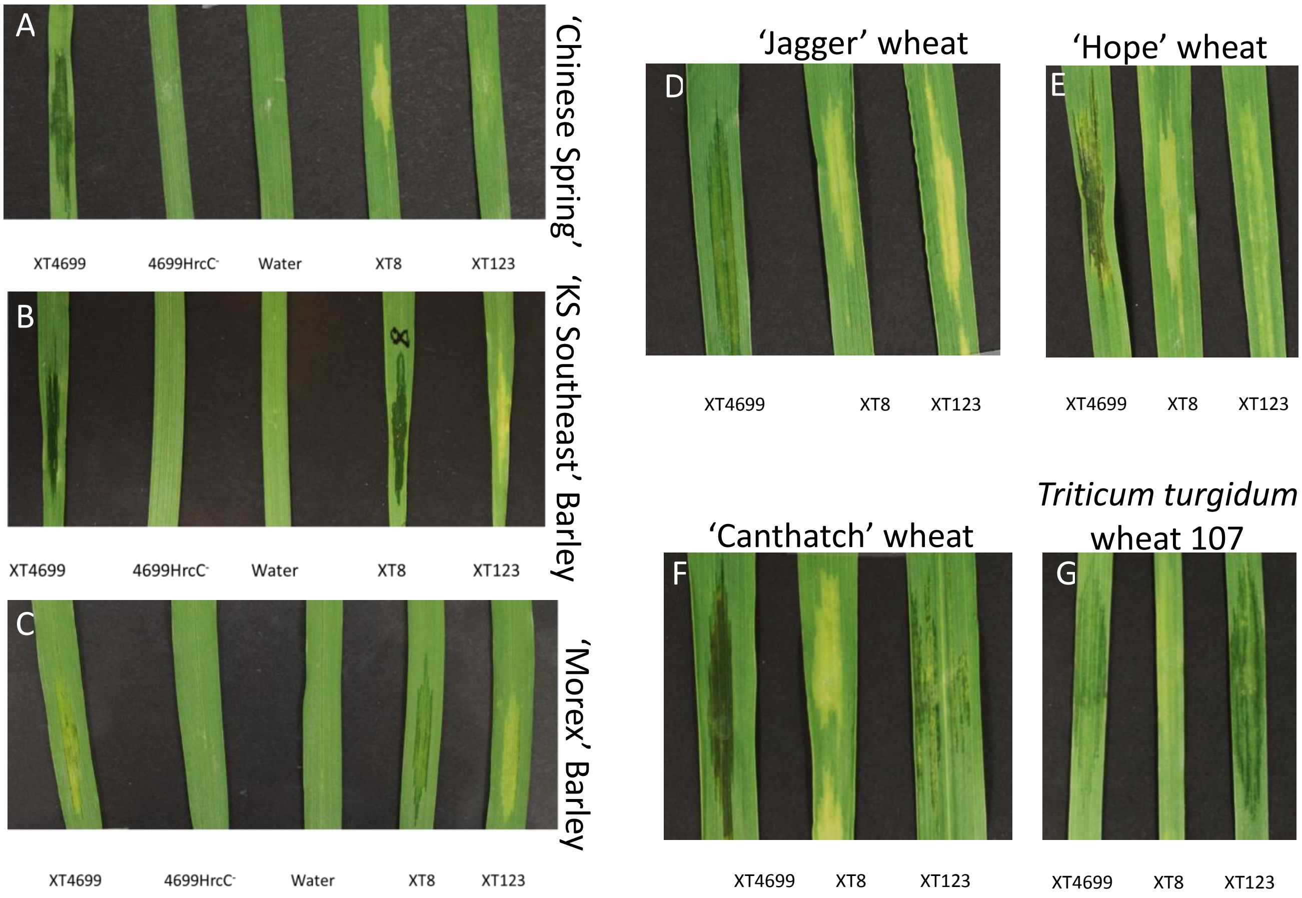

Figure S3. Different disease symptoms induced by pathovars of *X. translucens*. In A-G panels, different wheat and barley cultivars were inoculated. A, 'Chinese Spring' wheat; B, 'KS Southeast' barley; C, 'Morex' barley; D, 'Jagger' wheat; E, 'Hope' wheat; F, 'Canthatch' wheat; G, Triticum turgidum wheat #107. In A, B, C, plants are 3 weeks old and second leaves were inoculated. In D,E, F,G, leaves with similar age from 50-day-old plants were inoculated.
