# Supplementary material for: Long read and single molecule DNA sequencing simplifies genome assembly and TAL effector gene analysis of Xanthomonas translucens
Source: BMC Genomics. 2016 Jan 5;17:21. doi: 10.1186/s12864-015-2348-9 (PMC4700564; doi:10.1186/s12864-015-2348-9)
Supplement: Additional file 8: Figure S4. — Phylogenetic tree based on CRISPR Cas loci of X. translucens strains. The sequence of CRISPR Cas genes is annotated in RAST website http://rast.nmpdr.org. The phylogenetic tree was generated using Geneious software Version 6 with the Tamura-Nei genetic distance model and the Neighbor-joining method, with PXO99 CRISPR Cas genes as outgroup. The scale bar indicates number of nucleotide substitutions per site. (PDF 149 kb) [file 12864_2015_2348_MOESM8_ESM.pdf]

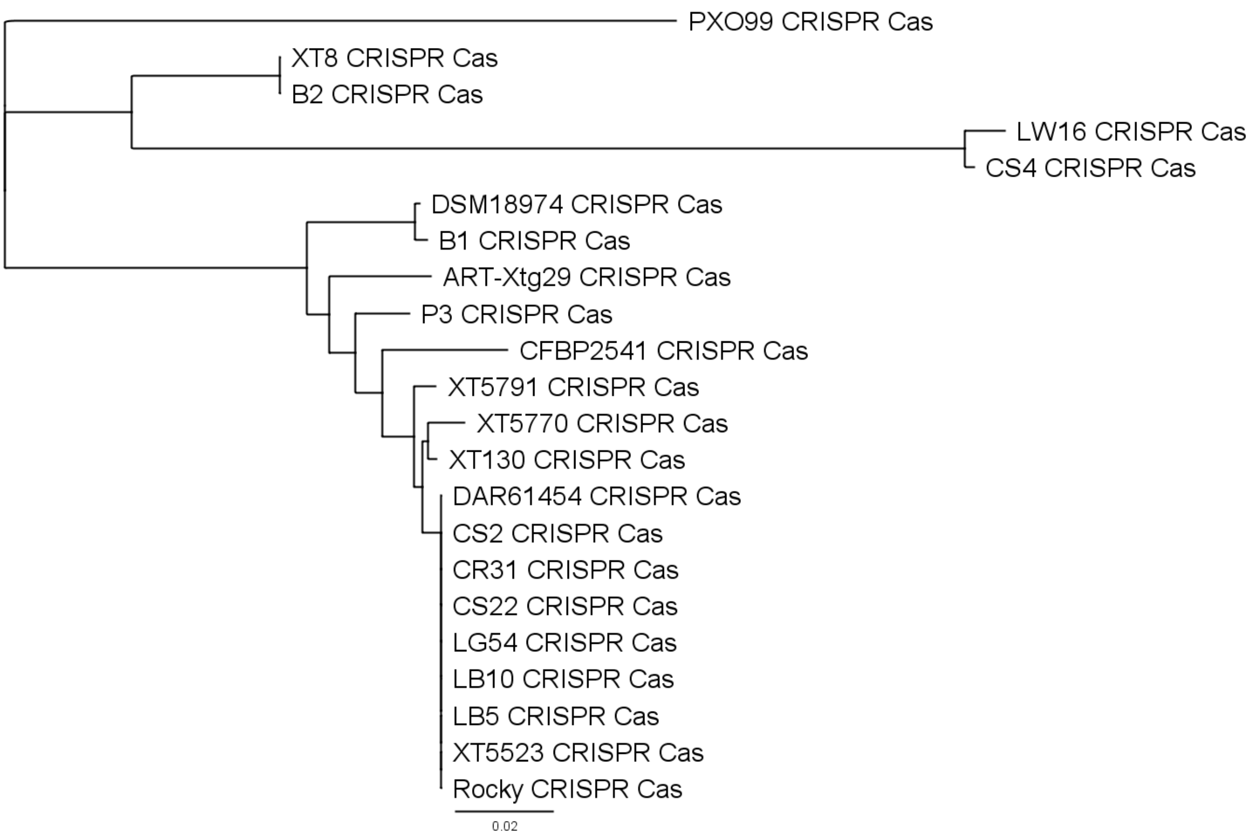

Figure S4. Phylogenetic tree based on CRISPR *Cas* loci of *X. translucens* strains. The sequence of CRISPR *Cas* genes is annotated in RAST website http://rast.nmpdr.org.The phylogenetic tree was generated using Geneious software Version 6 with the Tamura-Nei genetic distance model and the Neighbor-joining method, with PXO99 CRISPR *Cas* genes as outgroup. The scale bar indicates number of nucleotide substitutions per site.
