# Supplementary material for: Long read and single molecule DNA sequencing simplifies genome assembly and TAL effector gene analysis of Xanthomonas translucens
Source: BMC Genomics. 2016 Jan 5;17:21. doi: 10.1186/s12864-015-2348-9 (PMC4700564; doi:10.1186/s12864-015-2348-9)
Supplement: Additional file 9: Figure S5. — Syntenic gene clusters of type III secretion system among X. translucens strains. XT-Rocky and XT4699 are X. translucens pv. undulosa while ART-Xtg29 is X. translucens pv. gramins. White arrows (drawn to scale) represent orientation and position of Hrp genes. Black arrows indicate the two predicted IS elements in ART-Xtg29 strain. The names of Hrp genes are shown above or under the white arrows. The identity of homologous proteins of compared strains is shown between them. (PDF 155 kb) [file 12864_2015_2348_MOESM9_ESM.pdf]

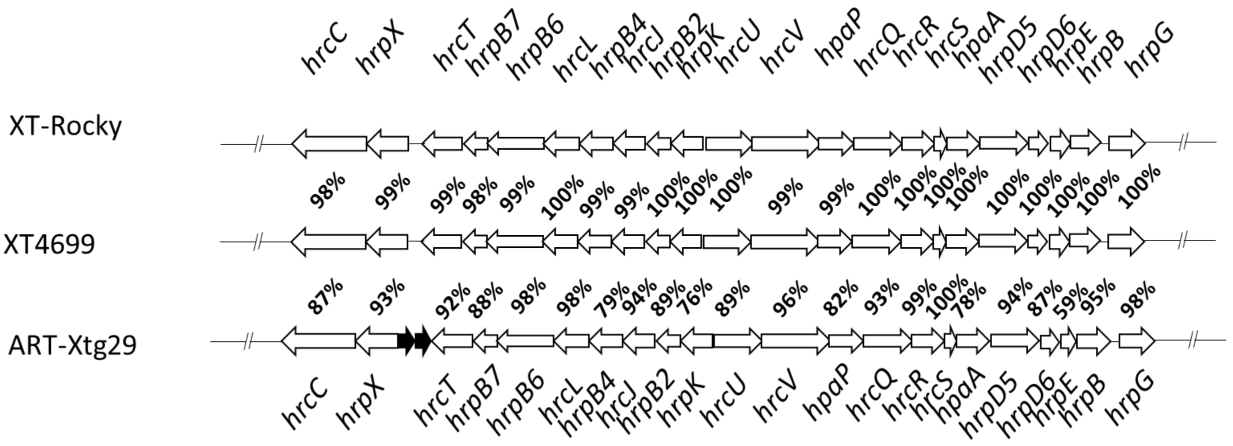

Figure S5. Syntenic gene clusters of type III secretion system among *X. translucens* strains. XT-Rocky and XT4699 are *X. translucens* pv. *undulosa* while ART-Xtg29 is *X. translucens* pv. *gramins*. White arrows (drawn to scale) represent orientation and position of Hrp genes. Black arrows indicate the two predicted IS elements in ART-Xtg29 strain. The names of Hrp genes are shown above or under the white arrows. The identity of homologous proteins of compared strains is shown between them.
