# Supplementary material for: Long read and single molecule DNA sequencing simplifies genome assembly and TAL effector gene analysis of Xanthomonas translucens
Source: BMC Genomics. 2016 Jan 5;17:21. doi: 10.1186/s12864-015-2348-9 (PMC4700564; doi:10.1186/s12864-015-2348-9)
Supplement: Additional file 10: Figure S6. — Bacterial population assay. Equal amount of 4x10^4^ dilution of bacterial inoculum of different tal mutants, hrcC mutant and WT were inoculated on 3-week-old Chinese Spring wheat by needleless syringe. At 6DPI, three inoculated leaves (3 cm) were pooled and ground together in each treatment. Samples were serially diluted and 100 μl of diluted samples were added to TSA plates. Plates were incubated at 28 °C for colony formation. Data represents the mean of Log10 CFU/cm leaf ± standard deviation. The * indicates the significant difference of bacterial population of hrcC − mutant compared to other tal mutants or WT at 6DPI under p-value <0.001 in the ANOVA statistics analysis. (PDF 157 kb) [file 12864_2015_2348_MOESM10_ESM.pdf]

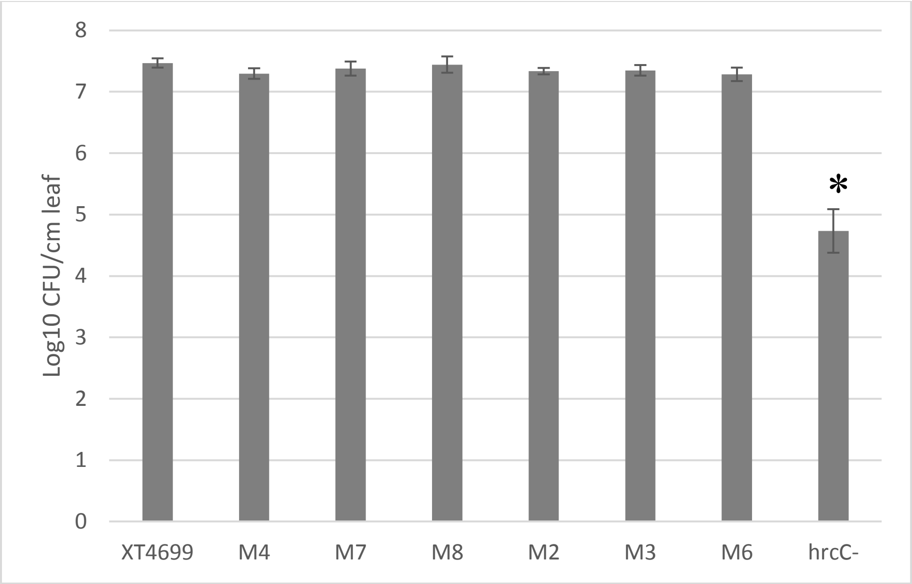

Figure S6. Bacterial population assay. Equal amount of $4x10^4$ dilution of bacterial inoculum of different *tal* mutants, *hrcC* mutant and WT were inoculated on 3-week-old Chinese Spring wheat by needleless syringe. At 6DPI, three inoculated leaves (3 cm) were pooled and ground together in each treatment. Samples were serially diluted and 100 µl of diluted samples were added to TSA plates. Plates were incubated at 28^0C for colony formation. Data represents the mean of Log10 CFU/cm leaf ± standard deviation. The * indicates the significant difference of bacterial population of *hrcC*$^-$ mutant compared to other *tal* mutants or WT at 6DPI under p-value <0.001 in the ANOVA statistics analysis.
