# Supplementary material for: Long read and single molecule DNA sequencing simplifies genome assembly and TAL effector gene analysis of Xanthomonas translucens
Source: BMC Genomics. 2016 Jan 5;17:21. doi: 10.1186/s12864-015-2348-9 (PMC4700564; doi:10.1186/s12864-015-2348-9)
Supplement: Additional file 11: Figure S7. — Southern blot analysis of two X. translucens pv. undulosa strains from Kansas. Genomic DNA digested by BamHI and hybridized by sphI fragment of TAL effector gene in X. oryzae pv. oryzae strain AXO1947. The size of blotted bands is consistent with assembly genome of XT4699. (PDF 182 kb) [file 12864_2015_2348_MOESM11_ESM.pdf]

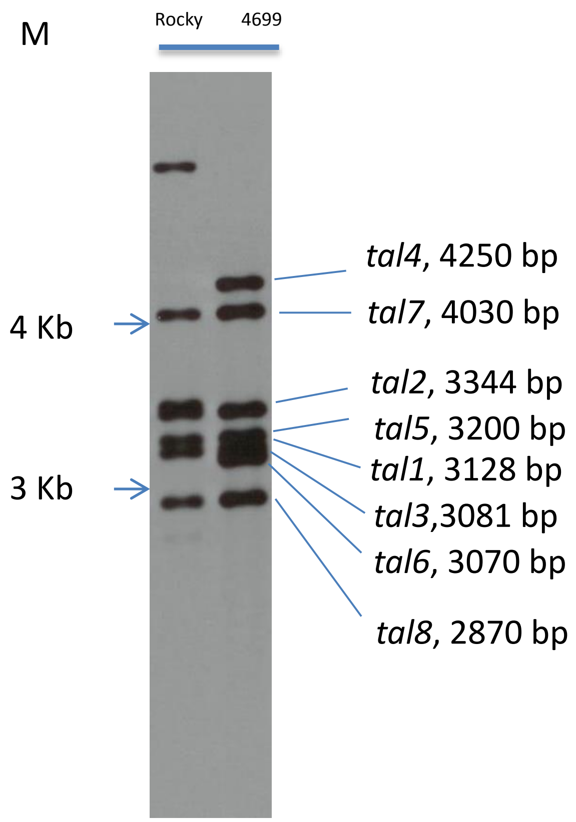

Figure S7. **Southern blot analysis of two *X. translucens* pv. *undulosa* strains from Kansas.** Genomic DNA digested by *Bam*HI and hybridized by *sph*I fragment of TAL effector gene in *X. oryzae* pv. *oryzae* strain AXO1947. The size of blotted bands is consistent with assembly genome of XT4699.
