# Supplementary material for: Long read and single molecule DNA sequencing simplifies genome assembly and TAL effector gene analysis of Xanthomonas translucens
Source: BMC Genomics. 2016 Jan 5;17:21. doi: 10.1186/s12864-015-2348-9 (PMC4700564; doi:10.1186/s12864-015-2348-9)
Supplement: Additional file 12: Table S5. — Amino acid sequences of TAL effector proteins in X. translucens pv. undulosa strain XT4699. (PDF 135 kb) [file 12864_2015_2348_MOESM12_ESM.pdf]

Additional file 12: Table S5. Amino acid sequences of TAL effector proteins in *X. translucens* pv. *undulosa* strain XT4699.

XT4699-Tal1
MDPIRSRTPSPARELQAGSQPDGVQPTADPRVSPPAGSPLDGLPARRTMSRTQLPPPPASGPSFSAGSFSDLLRQVDSSLFDAS<br>FFDSMPAFGAHHAQAATGELDEAQSALRAVDDPQPSASAAITAAPPRTKAAARRCSAQTLDALPAADVDLSTFGYSQQQQEKIK<br>PKVRSTVAQHHAALVGHGFTHAHIVELSKHPPALGTIAARYSEMIAALPEATHEDIVGVGKHCAGARTLEVLLMVVQELRAPPL<br>QLVTSQLLKIAKRGGVTAVEAVHASRNALTGAPLH<br>LLPDQVVAIVSHDGGKQSLETVQRLLPVLRQPPYG<br>LTPNQVVAIASYDGGKQSLETVERLLPVLCQPPYG<br>LTPNQVVAIASNIGGKPALETVQRLLPVLCQPPYG<br>LTPNQVVAIASNGGGKQALETVQRLLPVLCQEYG<br>LIPEQVVAIASNGGGKQALETVQRLLPVLCKEYG<br>LTPEQVVAIASNNGGKLALETVERLLPVLCQPPYG<br>LTPKQVVAIASYKGANQALGTVQRLLPVLCKPPYG<br>LTPDQVVAIASNGGAKQALETVERLLPVLCKPPYG<br>LTPDQVVAIASHDGGRQALETVQRLLPVLCQPPYG<br>LTPNQVVAIASNGGGKQALETVQRLLPVLCQEYG<br>LTRQQVVAIASNGGGKQSLETVQRLLPVLCKPPYN<br>LTPDQVVAIASNDGGKQALETVQRLLPVLCKPPYN<br>LTPEQVVAIASNGGGKQALETVQRLLPVLCKEYG<br>LTPEQVVAIASQDGGKQSLETVQRLLPVLCQLPYG<br>LTPNQVVAIASNHGGKQSLETVQRLLPVLCQPPYG<br>LTPNQVVAIASHDGGTQALE<br>SIFAQLSSPDPALAALTNDRLVALACIGGRPALDAVKKGLPHAPALITRVHNRVPEGTAHLVADLAQVVRVLSFFQCHSHPAQA<br>FDEAMRQFGMSRHGLLQLFRRVGVTELEAISGTLPPASQRWDRMLQASGRKGAKPPSASAQTQGQESLDAFADSLERELDAPSP<br>MHQAGQTLASSRKRSRSESSVNRSSAQQAAEVFVPEQRDAPPLLPLSSWGVKRRRTRIGGLPDPGTPTHGDLAASSAAFLEQDA<br>DPFAGAAEDFPAFDQEEIAWLKELLAH

XT4699-Tal2
MDPIRSRTPSPARELQAGSQPDGVQPTADPRVSPPAGSPLDGLPARRTMSRTQLPPPPASVPAFSAGSFSDLLRQVDSSLFDAS<br>FFDSMPAFGAHHAQAATGELDEVQSALRAADDPQPPVRVAVTAARPPRAKPAQRPRRAAQTSDASPAADVDLSTFGYSQQQQEK<br>IKPTVRSSVAQHHAALVGHGFTHAHIVELSKHPAALGTIAARYSEMIAALPEATHEDIVGVGKQWSGARALEALLMVAEELRAP<br>PLQLVTGQLLKIAKRGGVTAVEAVHASRNALTGAPLH<br>LTPDQVVAIVSNNGGKQALETVQRLLPVLCQPPYG<br>LTPEQVVAIASHDGAKPALETVQRLLPVLCQPPYG<br>LTPEQVVAIASNGGGKQALETVQRLLPVLCKEYG<br>LTPEQVVAIASNNGGKLALETVERLLPVLCQPPYG<br>LTPKQVVAIASHNGGKQALETVQRLLPVLCQPPYG<br>LTPEQVVAIASKGGGKQALETVQRLLPVLCQPPYG<br>LTPDQVVTIANNIGAKQALETVQRLLPVLCQPPYG<br>LTPNQVVAIASHDGAKQALETVQRLLPVLCQEYG<br>LTPGQVVAIANNIGGKPALETVQRLLPVLCQPPYN<br>LTPNQVVAIASNNGGKQALETVQRLLPVLCKPPHP<br>LTPNQVVAIASHDGAKQALETVQRLLPVLCKSPYG<br>LTPDQVVVIASHNGGKQALETVQRLLPVLCKPPYG<br>LTPNQVVAIASHDGGKPALETVQRLLPVLCQPPYG<br>LTPDQVVAIASHDGGKQALETVQRLLPVLCQDHG<br>LTPGQVVAIANNIGGKPALETVQRLLPVLCKEYG<br>LTPNQVVAIASHNGGKQALETVQRLLPVLCQPPYG<br>LTPEQVVAIASHDGGKPALETVQRLLPVLCQPPYG<br>LTPNQVVAIASQDGGTQALE<br>SIFAQLSSPDPALAALTNDRLVALACIGGRPALDAVKKGLPHAPALITRVHNRVPEGTAHLVADHAQVVRVLSFFQCHSQRGQV<br>FHEAMKRFEMSREGLLQLFRRVGVTELEAISGTLPPASQRWDRMLQASGRKGAKPPSASAQTQGQESLDAFADSLERELDAPSP<br>MHQAGQTLASNRKRSRSESSVNRSSAQQAAEVFVPEQRDAPPLLPLSSWGVKRRRTRIGGLPDPGTPTHGDLAASSAAFLEQDA<br>DPFAGAAEDFPAFDQEEIAWLKELLAH

XT4699-Tal3

MDPIRSRTPSPARELQAGSQPDAVQPIADRLVSPPAGSPLDGLPARRTMSRTQLPSPPASVPAFSAGSFSDLLRQVDSSLFDAS
FFDSMPAFGAHHAQAATGELDEVQSALRAADDPQPPVRVAVTAARPPRAKPAQRPRRAAQTSDASPAADVDLSTFGYSQQQQEK
IKPTVRSTVSQHHAALVGHGFTHAHIVELSKHPAALGTIAARYSEMIAALPEAAHEDIVGVGKQWSGARALEALLMVAEELRAP
PLQLVTGQLLKIAKGGGVTAVEAVHASRNALTGAPLH
LTPDQVVAIVSNNGGKQALETVQRLLPVLCQPPYN
LTPEQVVAIASHDGAKPALETVQRLLPVLCQPPYG
LTPEQVVAIASNGGGKQALEAVQRLLPVLCKEYG
LTPEQVVAIASHDGAKQALETVQRLLLVLCQEYG
LTPNQVVAIASHDGAKQALETVQRLLPVLCQPPYG
LTPNQVVAIASHNGGKQALETVQRLLPVLCQPPYG
LTPEQVVTIANNFGGKPALETVQRLLPVLCQPPYG
LTPNQVVAIANNIGAKPALETVQRLLPVLCQPPYG
LTPEQVVAIAGNHGAKQALETVQRLLPVLCQPPYG
LTPNQVVAIASHDGAKQALETVQRLLPVLCKPPHP
LTPNQVVAIASHDGAKQALETVQRLLPVLCKPPYG
LTPDQVVAIASHDGAKPALETVQRLLPVLCKPPYG
LTPDQVVAIASHNGGKQALETVQRLLPVLCQPSYG
LTRNQVVAIASHNGGKQALETVQRLLPVLCKEYG
LTPEQVVAIASHDGGTQALE
SIFAQLSSPDPALAALTNDRLVALACIGGRPALDAVKKGLPHAPALITRVHNRVPEGTAHLVADLAQVVRVLSFFQCHSHPAQA
FDEAMRQFGMSRHGLLQLFRRVGVTELEAISGTLPPASQRWDRMLQASGRKGAKPPSASAQTQGQESLDAFADSLERELDAPSP
MHQAGQTLASSRKRSRSESSVNRSSAQQAAEVFVPEQRDAPPLLPLSSWGVKRRRTRIGGLPDPGTPTHGDLAASSAAFLEQDA
DPFAGAAEDFPAFDQEEIAWLKELLAH

XT4699-Tal4

MEPIRSRTPSTARELQAGSQPDAVQPIADRLVSTPASSPLDGLPARRMVSRTSPPPSPARSPAFSAGSLSGLLRQQIDPSLFAG
SPFDSLPSFGAARAESAPGEGDEVQSGLRAVDDPQPSASAAITAAPPRTKAAARRRSAQTSDALPAAHVDLGTFGYSQQQQEKI
KPKVRSTLAQHHEALVGHGFTHAHIVELSKHPPALGTIAARYSEMIAALPEATHEDIVGVGKQKSGARALEALLTVAEELRAPP
LQLVTGQLLKIAKRGGVNAVEAVHASRNALTGAPLH
LLPDQVVAIVSNHGSKLALGTVQRLLPVLCKPPYG
LTRNQVVAIVNNNGGKQALETVHRLLPVLCQPPYG
LTPEQVVAIASHDGGRQALETVHRLLPVLRQPPYG
LTLEQVVAIASNNGGKQALETVQRLLPVLCQPPYG
LTPDQVVTIASHDGGRQALETVQRLLPVLCQPPYG
LTPNQVVAIASNHGGKQALETVQRLLPVLCQPLHG
LTPDQVVTIASHDGGRQALETVQRLLPVLCQPPYG
LTPKQVVAIANYKGAKQALETVQRLLPLLCKPPYG
LTPDQVIAIVSNGGGKPALETVRRLLPVLCKHPYG
LTPKQVVAIVSNHGGKPALETVRRLLPVLCKHPYG
LTPKQVVAIASY*GGKPALETVQRLLPVLCQPPYG
LTPNQVVAIASHDGGRQALETVQRLLPVLRQPPYG
LTPDQVVAIASNNGGKQALETVQRLLPVLCQPPYS
LIPDQVVAIASNIGAKQALETVQRLLPVLCQPPYG
LTTDQVIAIASNGGSKQALETVERLLPVLCQPPYG
LTPDQVVAIASQDGGKQALE
SIFAQLSSPDPALAALTNDRLVALACLGGRPALDAVKKGLPHAPALVTRTHNRVPEGTAHLVADHAQVVRVLGFFQCHSHPAQA
FHEAMTRFEMSREGLLQLFRRVGVTELEAISGTLPPASQRWHRILQALGVKGAKPSSASAQTPSQESVDAFADSLERELDAPSP
IHEADRARASNRKRSRSESSVNRSSAQQAAEVFVPEQRDAPPLLPLSSWGVKRQRTRIGGLPDPGMPTDGELAASSAAFLEQDA
DPFAGAAEDFPVFDQEEIAWLMTLLPH

XT4699-Tal5
MDPIRSRTPSPARELQAGSQPDAVQPIADRLVSPPAGSPLDGLPARRTMSRTQLPSPPASVPAFSAGSFSDLLRQVDSSLFDAS
FFDSMPAFGAHHAQAATGELDEVQSALRAADDPQPPVRVAVTAARPPRAKPAQRPRRAAQTSDASPAADVDLSTFGYSQQQQEK
IKPTVRSTVAQHHAALVGHGFTHAHIVELSKHPPALGTIAARYSEMIAALPEATHEDIVGVGKQWSGARALDALLMVAEELRAP
PLQLVTGQLLKIAKRGGVTAVEAVHASRNALTGAPLH
LTPDQVVAIVSNNGGKQALETVERLLPVLCQPPYG
LTPEQVVAIASHDGAKPALETVQRLLPVLCQPPYG
LTPEQVVAIASNGGGKPALETVQRLLPVLCKEYG
LTPEQVVAIASNNGGKLALETVERLLPVLCQPPYG
LTPNQVVAIASHNGGKQSLETVQRLLPVLCQPPYG
LTPEQVVAIASHNGGKQALETVQRLLPVLCQPPYG
LTPEQVVTIANNIGGKQALETVQRLLPVLCQPPYG
LTPNQVVAIASNIGAKPALETVQRLLPVLCQPPYG
LTPEQVVTIANNIGGKPALETVQRLLPVLRKPPYG
LTPEQVVAIASNHGGKQALETVQRLLPVLCKPPYG
LTPNQVVAIASNNGAKQALETVQRLLPVLCKPPHP
LTPNQVVAIASHDGAKQALETVQRLLPVLCQPPYG
LTPEQVVVIASNNGGKQALETVQRLLPVLCQPSYG
LTPEQVVAIASNHGGKQALETVQRLLPVLCQPPYG
LTPNQVVAIASHDGGKPALETVQRLLPVLCQPPYG
LTPNQVVAIASHDGGTQALE
SIFAQLSSPDPALAALTNDRLVALACIGGRPALDAVKKGLPHAPALITRVHNRVPEGTAHLVADLAQVVRVLSFFQCHSHPAQA
FDEAMRQFGMSRHGLLQLFRRVGVTELEAISGTLPPASQRWDRMLQASGRKGAKPSSASAQTPSQESVDAFADSLERELDAPSP
MHQAGQTLASSRKRSRSESSVNRSSAQQAAEVFVPEQRDAPPLLPLSSWGVKRRRTRIGGLPDPGTPTHGDLAASSGAFLEQDA
DPFAGAAEDFPAFDQEEIAWLKELLAH

XT4699-Tal6
MDPIRSRTPSPARELQAGSQPDAVQPIADRLVSPPAGSPLDGLPARRTMSRTQLPSPPASVPAFSAGSFSDLLRQVDSSLFDAS
FFDSMPAFGAHHAQAATGELDEVQSALRAADDPQPPVRVAVTAARPPRAKPAQRPRRAAQTSDASPAADVDLSTFGYSQQQQEK
IKPTVRSTVAQHHAALVGHGFTHAHIVELSKHPAALGTIAARYSEMIAALPEATHEDIVGVGKQWSGARALETLLMVAEELRAP
PLQLVTGQLLKIAKGGGVTAVEAVHASRNALTGAPLH
LTPDQVVAIVSHDGGKQSLETVQRLLPVLCQPPYG
LTPNQVVAIASHNGGKQALETVQRLLPVLCQEYG
LTPEQVVAIASHNGGKQALETVQRLLPVLCQEYG
LTPEQVVAIASHDGAKQALETVQRLLPVLCQPPYG
LTPEQVVAIASNHGGKLALETVERLLPVLCQPPYG
LTPNQVVAIASNHGGKQALETVQPLLPVLCQPPYG
LTPEQVVAIASHGGAKQALKTVQRLLPVLCQDHG
LTPEQVVAIANHDGAKQALETVQRLLPVLCQPPYG
LTPEQVVAIASKGGGKQALETVQRLLPVLCQPPYG
LTPDQVVTIASNNGGKPALETVRRLLPVLCKPPYG
LTPKQVVAIASY*GGKQALETVQRLLPVLCKPPYG
LTPEQVVAIASNGGGKPALETVQRLLPVLCQEYG
LTPNQVVAIASHDGAKPALETVQRLLPVLCQEYG
LTPNQVVAIASHDGGKPALETVQRLLPVLCQPPYG
LTPEQVVAIASHNGGTQALE
SIFAQLSSPDPALAALTNDRLVALACIGGRPALDAVKKGLPHAPALITRVHNRVPEGTAHLVADLAQVVRVLSFFQCHSHPAQA
FDEAMRQFGMSRHGLLQLFRRVGVTELEAISGTLPPASQRWDRMLQASGRKAAKPPSASAQTQGQESLDAFADSLERELDAPSP
MHQAGQTLASSRKRSRSESSVNRSSAQQAAEVFVPEQRDAPPLLPLSSWGVKRRRTRIGGLPDPGTPTHGDLAASSAAFLEQDA
DPFAGAAEDFPAFDQEEIAWLKELLAH

XT4699-Tal7
MDPIRSRTPSPARELLAGSQPDGVQPTADPRVSPPAGSPLDGLPARRTMSRTQLPPPSASGPAFSAGSFSDLLRQVDSSLFNAS
LFDSMPAFGAHHAQAATGELDEAQSALRAVDDPQSSASAAITAAPPRTKAAARRRSAQTLDASPAADVDLSTFGYSQQQQEKIK
PTVRSSVAQHHAALVGHGFTHAHIVELSKHPAALGTIAARYSEMIAALPEATHEDIVGVGKQWSGARALEALLMVAEELRAPPL
QLVTGQLLKIAKRGGVTAVEAVHASRNALTGAPLH
LTPDQVVAIVSHNGGKQALETVQRLLPVLCQPPYN
LTPEQVVAIASHDGGKQALETVQRLLLVLCQECG
LTPNQVVAIASHDGAKPALETVHRLLPVLCQPPYG
LTPNQVVAIASHDGAKQALETVQRLLPVLCQDHG
LTPGQVVAIADNIGGKQALETVQRLLPVLCKPPYG
LTPEQVVTIANNIGGKPALETVQRLLPVLCKPPYG
LTPEQVVTIANNIGAKPALETVHRLLPVLRKPPYG
LTPEQVVAIASHNGAKPALETVQRLLPVLCQPPYG
LTPNQVVAIASHDGAKQALETVQRLLPVLCKPPHP
LTPNQVVAIASHDGAKQALETVQRLLPVLCQPPYG
LTPDQVVVIASNNGGKQALETVQRLLPVLCQPPYG
LTPEQVVAIASNNGGKPALETVQRLLPVLCQPPYD
LTPDQVVAIANNIGAKPALETVQRLLPVLCQPPYG
LTPEQVVAIASNNGGKQALETVQRLLPVLCQPPYG
LTPYQVVAIASHDGGTQALE
SIFAQLSSPDPALATLTNDRLVALACIGGRPALDAVKKGLPHAPELITRVHNRVPEGTAHLVADHAQVVRVLGFFQCHSQRGQV
FHEAMKRFEMSREGLLQLFRRVGVTELEAISGTLPPASQRWDRMLQASGRKGAKPPSASAQTQGQESLDAFADSLERELDAPSP
MHQAGQTLASSRKRSRSESSVNRSSAQQAAEVFVPEQRDAPLLLPLSSWGVKRRRTRIGGLPDTGMPTDGELAASSAAFLEQDA
DPFAGAAEDFPAFDQEEIAWLRELLAH

XT4699-Tal8
MDPIRSRTPSPARELQAGSQPDAVQPIADRLVSPPAGSPLDGLPARRTMSRTQLPSPPASVPAFSAGSFSDLLRQVDSSLFDAS
FFDSMPAFGAHHAQAATGELDEVQSALRAADDPQPPVRVAVTAARPPRAKPAQRPRRAAQTSDASPAADVDLSTFGYSQQQQEK
IKPTVRSTVAQHHAALVGHGFTHAHIVELSKHPAALGTIAARYSEMIAALPEATHEDIVGVGKQWSGARALEALLMVAEELRAP
PLQLVTGQLLKIAKRGGVTAVEAVHASRNALTGAPLH
LTPDQVVAIVSNNGGKQALETVQRLLPVLCKPPYG
LTPEQVVAIASNGGGKPALETVQRLLPVLCQEYG
LTPNQVVAIASHDGAKQALETVQRLLPVLCQPPHP
LTPNQVVAIASHDGGKPALETVQRLLPVLCKPPYG
LTPEQVVAIANHDGAKQALETVQRLLPVLCQPPYG
LTPEQVVAIASKGGGKQALETVQRLLPVLCQPPYG
LTPDQVVTIASNNGGKPALETVRRLLPVLCKPPYG
LTPKQVVAIASY*GGKQSLETVQRLLPVLCKPPYG
LTPEQVVAIASNGGGKPALETVQRLLPVLCQEYG
LTPNQVVAIASHDGAKPALETVQRLLPVLCQEYG
LTPNQVVAIASHDGAKQALETVQRLLPVLCKPPYG
LTPEQVVAIASQDGGKQSLETVQRLLPVLCKPPYG
LTPEQVVAIASHNGGTQALE
SIFAQLSSPDPALAALTNGRLVALACIGGRPALDAVKKGLPHAPALITRVHNRVPEGTAHLVADLAQVVRVLSFFQCHSHPAQA
FDEAMRQFGMSRHGLLQLFRRVGVTELEAISGTLPPASQRWDRMLQASGRKGAKPSSASAQTPSQESVDAFADSLERELDAHSP
MHQAGQTLASSRKRSRSESSVNRSSAQQAAEVFVPEQRDAPPLLPLSSWGVKRRRTRIGGLPDPGTPTHGDLAASSAAFLEQDA
DPFAGAAEDFPAFDQEEIAWLKELLAH

Note: The 34-amino-acid repeats, due to variation in the 32nd-35th amino acids of repeats, are marked by gray color. The 12th and 13th amino acids of each repeat are variable and underlined. The absence of 13th amino acid in repeats is indicated by *.
