# Supplementary figures and images for: Long read and single molecule DNA sequencing simplifies genome assembly and TAL effector gene analysis of Xanthomonas translucens
Source: BMC Genomics. 2016 Jan 5;17:21. doi: 10.1186/s12864-015-2348-9 (PMC4700564; doi:10.1186/s12864-015-2348-9)

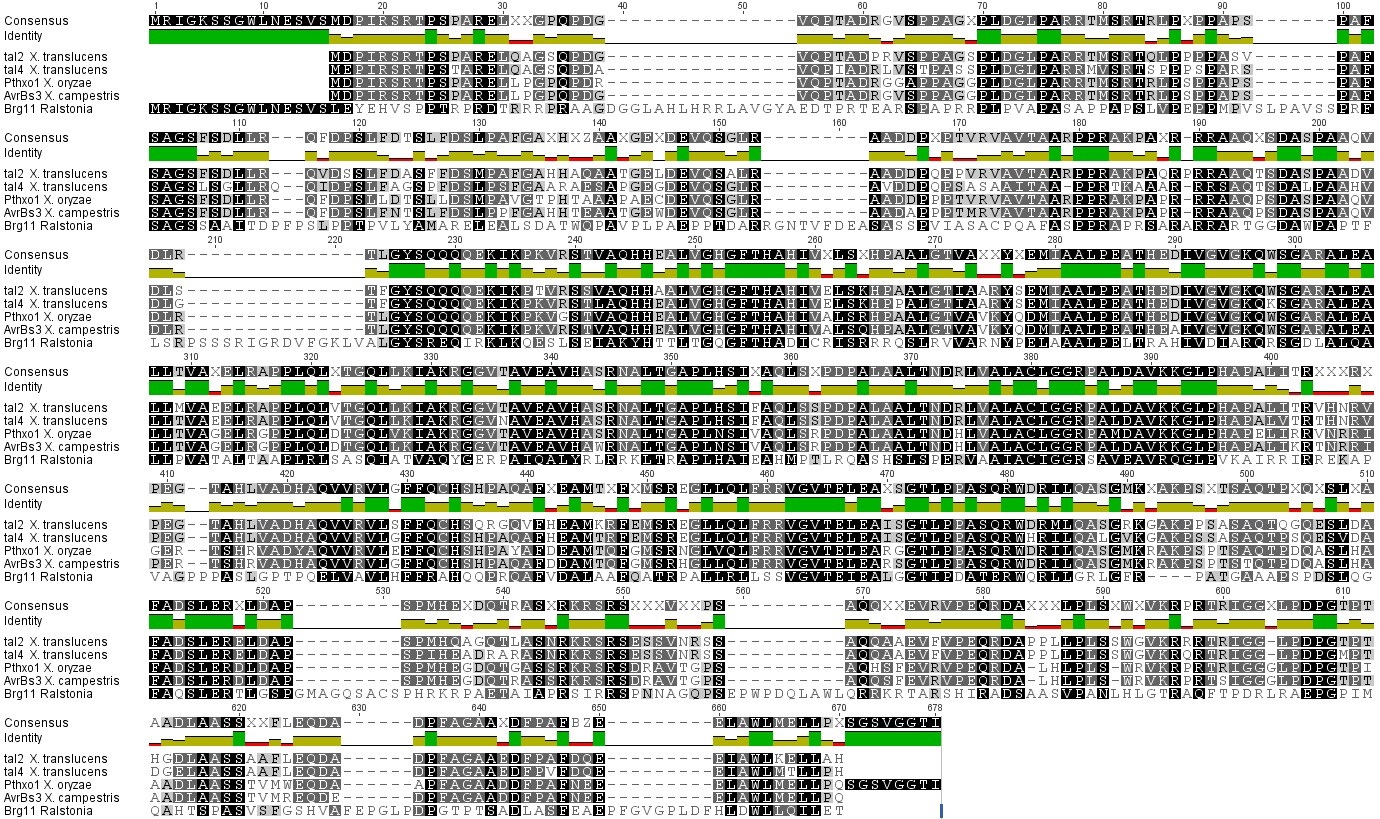

Supplement: Additional file 13: Figure S8. — Alignment of concatenated N-terminal and C-terminal amino acid sequences of TAL effectors. The sequences of Tal2 and Tal4 of X. translucens XT4699, PthXo1 of X. oryzae PXO99, AvrBs3 of X. campestris 85–10 and Brg11 of Ralstonia Solanacearum GMI1000 are applied for alignment, which is generated by Geneious software. (JPG 737 kb) [file 12864_2015_2348_MOESM13_ESM.jpg]
