# Supplementary material for: Long read and single molecule DNA sequencing simplifies genome assembly and TAL effector gene analysis of Xanthomonas translucens
Source: BMC Genomics. 2016 Jan 5;17:21. doi: 10.1186/s12864-015-2348-9 (PMC4700564; doi:10.1186/s12864-015-2348-9)
Supplement: Additional file 14: Figure S9. — Most TAL effector genes are flanked by transposon elements. The blue arrows indicate the TAL effector genes and the dark red arrows show the transposase genes; the gray arrows indicate other genes. The genes are listed as following: 1, RpoD, encoding RNA polymerase subunit sigma-70; 2, Hypothetical genes; 3, Acetyltransferase and hypothetical genes; 4, Type III effector gene xopL; 5, vgrG, encoding type IV secretion protein. (PDF 168 kb) [file 12864_2015_2348_MOESM14_ESM.pdf]

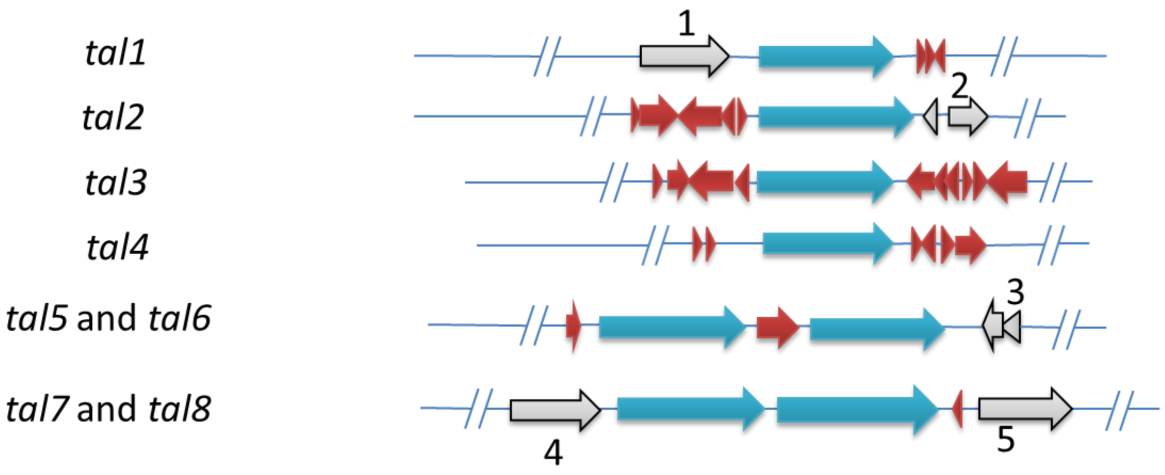

Figure S9. Most TAL effector genes are flanked by transposon elements. The blue arrows indicate the TAL effector genes and the dark red arrows show the transposase genes; the gray arrows indicate other genes. The genes are listed as following: 1,*RpoD*, encoding RNA polymerase subunit sigma-70; 2, Hypothetical genes; 3, Acetyltransferase and hypothetical genes; 4,Type III effector gene *xopL*;5, *vgrG*, encoding type IV secretion protein.
