# Supplementary material for: Long read and single molecule DNA sequencing simplifies genome assembly and TAL effector gene analysis of Xanthomonas translucens
Source: BMC Genomics. 2016 Jan 5;17:21. doi: 10.1186/s12864-015-2348-9 (PMC4700564; doi:10.1186/s12864-015-2348-9)
Supplement: Additional file 15: Figure S10. — XT4699-tal7 is a conserved TAL effector gene. Primer pair used for cloning tal7 gene in XT4699 was applied in PCR with template DNA of other strains from North Dakota and Kansas. PCR bands with similar size as XT4699-tal7 were shown in the blue box. (PDF 118 kb) [file 12864_2015_2348_MOESM15_ESM.pdf]

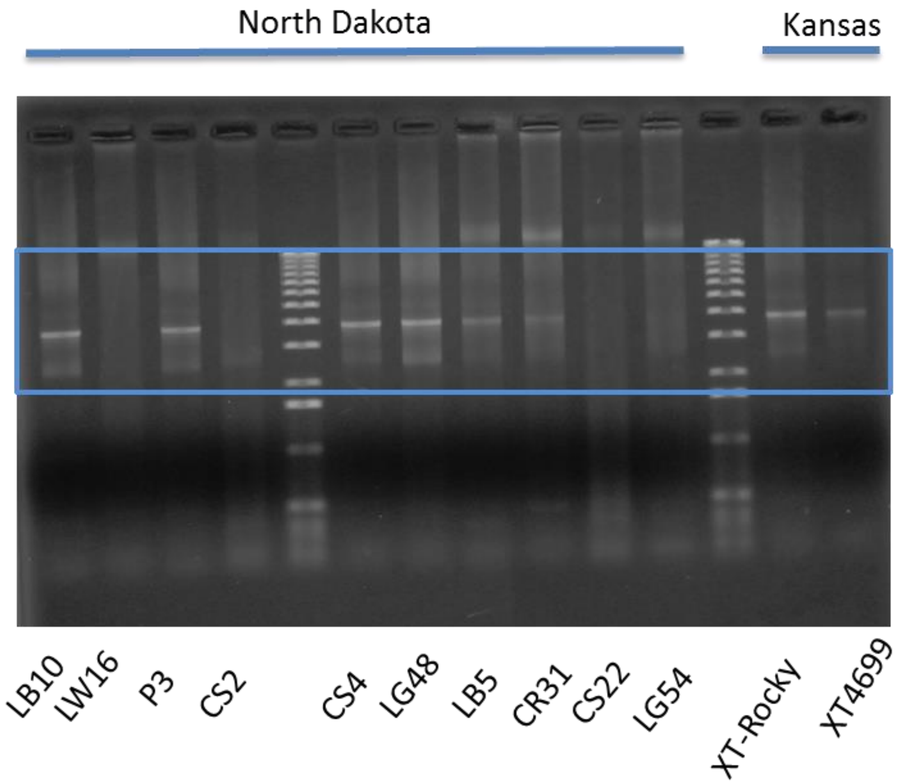

Figure S10. XT4699-*tal7* is a conserved TAL effector gene. Primer pair used for cloning *tal7* gene in XT4699 was applied in PCR with template DNA of other strains from North Dakota and Kansas. PCR bands with similar size as XT4699-*tal7* were shown in the blue box.
