# Supplementary material for: Long read and single molecule DNA sequencing simplifies genome assembly and TAL effector gene analysis of Xanthomonas translucens
Source: BMC Genomics. 2016 Jan 5;17:21. doi: 10.1186/s12864-015-2348-9 (PMC4700564; doi:10.1186/s12864-015-2348-9)
Supplement: Additional file 17: Figure S11. — Validation of all eight TAL mutants in XT4699 by PCR. The sequence of specific primers for each TAL gene and the primer from the vector are provided in Table S6. The desired PCR product size is around 1.5 kb. The corresponding names of TAL genes are shown for all 8 mutants. A, M1 (tal2 mutant), M2 (tal6 mutant); B, M3 (tal7 mutant), M4 (tal1 mutant); C, M5 (tal5 mutant), M6 (tal8 mutant); D, M7 (tal3 mutant), M8 (tal4 mutant). (PDF 351 kb) [file 12864_2015_2348_MOESM17_ESM.pdf]

A

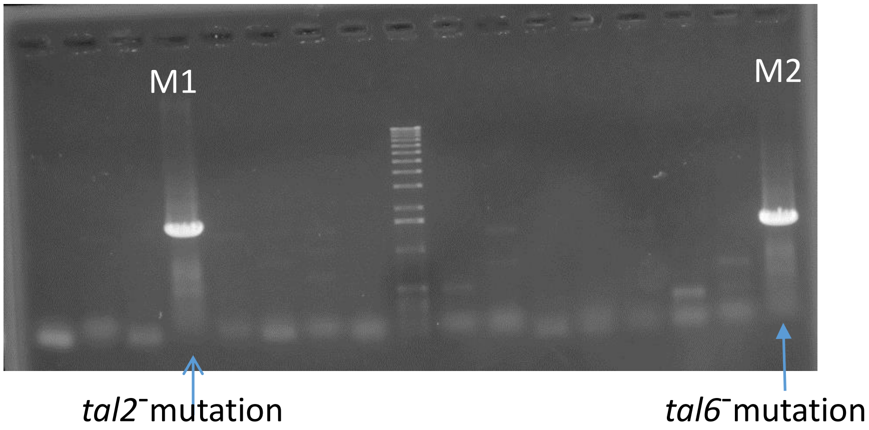
M1
M2
tal2⁻ mutation
tal6⁻ mutation

B

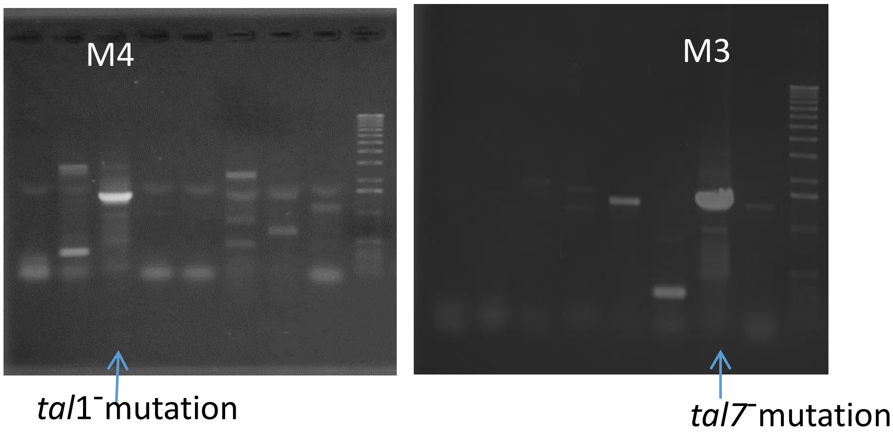
M4
M3
*tal*1⁻ mutation
tal7⁻ mutation

C

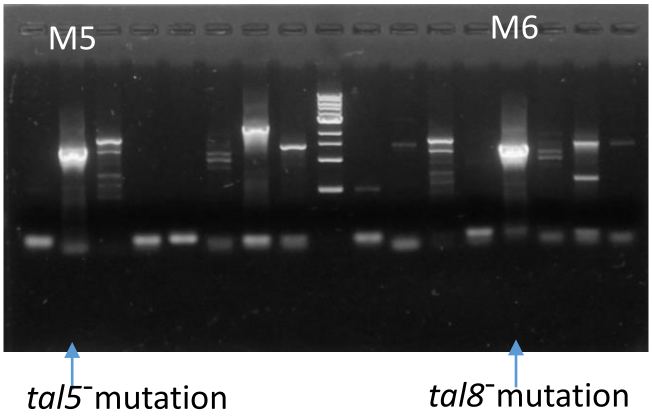
M5
M6
tal5⁻ mutation
tal8⁻ mutation

D

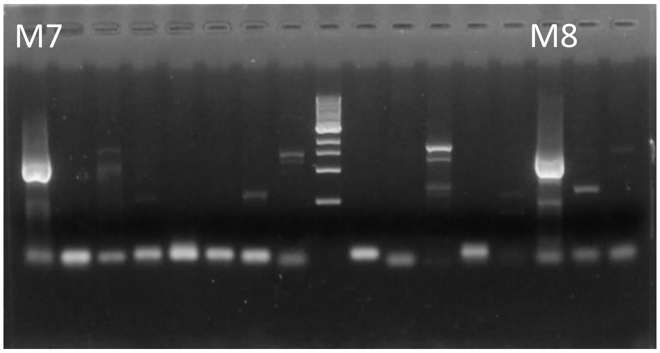

tal3^{-}mutation *tal4*$^{-}$mutation

Figure S11. Validation of all eight TAL mutants in XT4699 by PCR. The sequence of specific primers for each TAL gene and the primer from the vector are provided in Table S6. The desired PCR product size is around 1.5 kb. The corresponding names of TAL genes are shown for all 8 mutants. A, M1 (*tal2* mutant), M2 (*tal6* mutant); B, M3 (*tal7* mutant), M4 (*tal1* mutant); C, M5 (*tal5* mutant), M6 (*tal8* mutant); D, M7 (*tal3* mutant), M8 (*tal4* mutant)
