# Supplementary material for: Long read and single molecule DNA sequencing simplifies genome assembly and TAL effector gene analysis of Xanthomonas translucens
Source: BMC Genomics. 2016 Jan 5;17:21. doi: 10.1186/s12864-015-2348-9 (PMC4700564; doi:10.1186/s12864-015-2348-9)
Supplement: Additional file 18: Figure S12. — Induction of host genes is impaired by mutation of specific TAL genes. M2 is lack of tal6, M3 is lack of tal7 and M4 is the mutant of tal1. A, relative expression level of Ta.7291.1.S1_at, corresponding to succinate dehydrogenase subunit gene, was calculated compared to M2 treatment; B, relative expression level of Ta.14824.1.S1_at, corresponding to choline transporter related gene, was calculated compared to M3 treatment; C, relative expression level of Ta.9765.1.S1_at, corresponding to cell wall invertase gene, was calculated compared to M4 treatment. 2-∆∆Ct method was applied in calculation. The * indicates significant difference compared to other TAL mutants treated samples with P-value < 0.05 in the ANOVA statistics analysis. (PDF 191 kb) [file 12864_2015_2348_MOESM18_ESM.pdf]

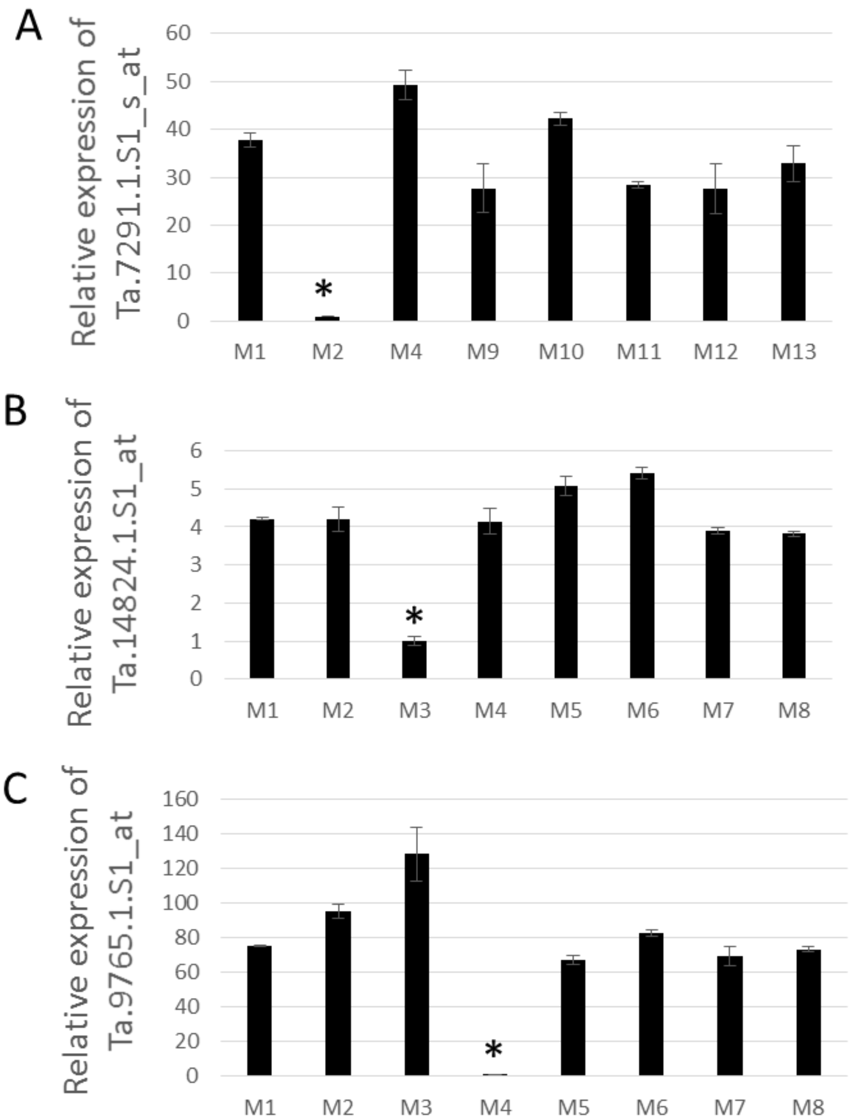

Figure S12. Induction of host genes is impaired by mutation of specific TAL genes.

M2 is lack of *tal6*, M3 is lack of *tal7* and M4 is the mutant of *tal1*. A, relative expression level of Ta.7291.1.S1_at, corresponding to succinate dehydrogenase subunit gene, was calculated compared to M2 treatment; B, relative expression level of Ta.14824.1.S1_at, corresponding to choline transporter related gene, was calculated compared to M3 treatment; C, relative expression level of Ta.9765.1.S1_at, corresponding to cell wall invertase gene, was calculated compared to M4 treatment. $2^{-\Delta\Delta Ct}$ method was applied in calculation. The * indicates significant difference compared to other TAL mutants treated samples with P-value<0.05 in the ANOVA statistics analysis.
