# Supplementary material for: Long read and single molecule DNA sequencing simplifies genome assembly and TAL effector gene analysis of Xanthomonas translucens
Source: BMC Genomics. 2016 Jan 5;17:21. doi: 10.1186/s12864-015-2348-9 (PMC4700564; doi:10.1186/s12864-015-2348-9)
Supplement: Additional file 19: Figure S13. — Induction of Ta.14164.1.S1_s_at and Ta.7291.1.S1_s_at is strain specific. XT8 is X. translucens pv. translucens, XT123 is X. translucens pv. cerealis, XT130, XT5523, XT5770 and XT5791 are strains of X. translucens pv. undulosa. M2 is the mutant of XT4699 lack of tal6. The relative expression level is calculated relative to M2 treatment by with 2-∆∆Ct method. A, the relative expression of Ta.14164.1.S1_s_at, corresponding to bHLH gene, was calculated; B, the relative expression of Ta.7291.1.S1_s_at, corresponding to succinate dehydrogenase gene, was calculated. The lowercase letters indicate significantly different groups with P-value < 0.05 in the ANOVA statistics analysis. (PDF 178 kb) [file 12864_2015_2348_MOESM19_ESM.pdf]

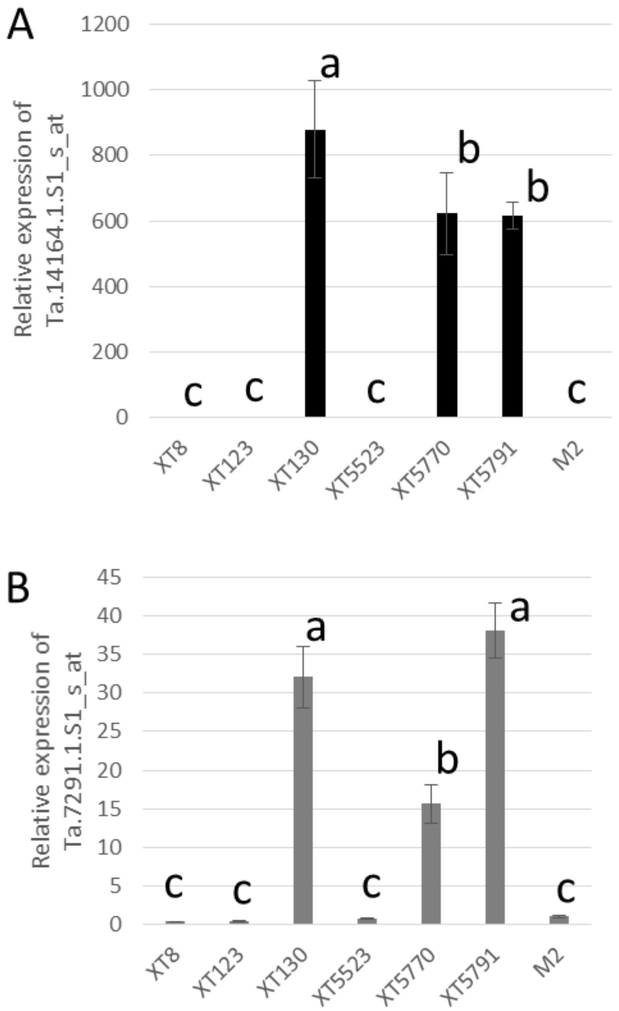

Figure S13. Induction of Ta.14164.1.S1_s_at and Ta.7291.1.S1_s_at is strain specific. XT8 is *X. translucens* pv. *translucens*, XT123 is *X. translucens* pv. *cerealis*, XT130, XT5523, XT5770 and XT5791 are strains of *X. translucens* pv. *undulosa*. M2 is the mutant of XT4699 lack of *tal6*. The relative expression level is calculated relative to M2 treatment by with $2^{-\Delta\Delta Ct}$ method. A, the relative expression of Ta.14164.1.S1_s_at, corresponding to *bHLH* gene, was calculated; B, the relative expression of Ta.7291.1.S1_s_at, corresponding to succinate dehydrogenase gene, was calculated. The lowercase letters indicate significantly different groups with P-value<0.05 in the ANOVA statistics analysis.
